# Nitrogen‐Doped Borane Cluster Network for High‐Performance Supercapacitors Under Universal pH Conditions

**DOI:** 10.1002/cssc.202502009

**Published:** 2026-01-29

**Authors:** Abhishek Udnoor, Samikannu Prabu, Madhan Vinu, Matouš Kloda, Andrii Mahun, Libor Kobera, Michael G. S. Londesborough, Kung‐Yuh Chiang, Jan Demel

**Affiliations:** ^1^ Department of Materials Chemistry Institute of Inorganic Chemistry of the Czech Academy of Sciences Řež Czech Republic; ^2^ Graduate Institute of Environmental Engineering National Central University Tao‐Yuan City Taiwan; ^3^ Department of Structural Analysis Institute of Macromolecular Chemistry of the Czech Academy of Sciences Prague Czech Republic

**Keywords:** borane cluster network, N doping, porous polymer, solid state NMR, supercapacitors

## Abstract

Supercapacitors have garnered considerable attention as next‐generation energy storage systems due to their high‐power density, rapid charge–discharge kinetics, and long operational lifespan. In this study, we report the design and development of a nitrogen‐doped activated borane (**ActB**), a porous borane cluster‐based network, synthesized through the controlled cothermolysis of *arachno*‐B_9_H_13_(NEt_3_) and [Et_3_NH][*nido*‐B_11_H_14_] in toluene. The resulting polymeric materials integrate electron‐rich nitrogen sites with the unique 3D boron cluster architecture, offering a synergistic platform for enhanced electrochemical performance. Electrochemical evaluation in a three‐electrode system revealed a high specific capacitance of 607 F g^−1^ at 0.5 A g^−1^, with remarkable cycling stability, retaining 95% of the initial capacitance after 15,000 charge–discharge cycles. When configured into an asymmetric supercapacitor device using activated carbon as the negative electrode, the system achieved a specific capacitance of 354 F g^−1^, along with an energy density of 25.6 Wh kg^−1^ and a power density of 486.2 W kg^−1^ at a current density of 0.5 A g^−1^. The device also demonstrated long‐term reliability, retaining 88% of its initial capacitance after 15,000 cycles. The outstanding performance is attributed to the integration of redox‐active nitrogen functionalities and the inherent stability and tunability of the borane‐based framework. This work establishes nitrogen‐doped borane cluster polymers as a promising new class of electrode materials for high‐performance supercapacitors and broader electrochemical energy storage applications.

## Graphical abstract

Nitrogen‐doped borane cluster network was prepared as a porous electrode material, featuring 1–2 nm micropores and specific surface area up to 626 m_2_ g_−1_. It delivers outstanding supercapacitor performance, reaching 607 F g_−1_ in a three‐electrode configuration and 354 F g_−1_ in an asymmetric supercapacitor device.

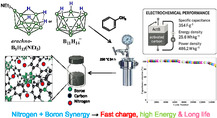

## 1 Introduction

As energy storage devices, supercapacitors (SC), also referred to as ultracapacitors or electrochemical capacitors, have attracted a lot of interest because of their high‐power density, quick charge and discharge times, and extended cycle life [1, 2, 3, 4]. However, when compared with traditional batteries, their energy density is still lagging. To address this, significant resources are being dedicated to the development of novel materials aimed at enhancing their capacitance. Among the many materials tested, those based on C, N, O, S, and B have shown particularly promising results. Doping is a powerful technique for enhancing the electrochemical properties of materials. It can preserve the porous structure of the material's original scaffold whilst facilitating the quick transit of ions through it, thus improving charge storage properties and raising the total energy and power density of SCs [5, 6, 7, 8, 9, 10].

In recent years, significant effort has been devoted to the development of novel porous polymers containing heteroatoms [11, 12, 13, 14], novel building units [15, 16, 17, 18], or tailored functionalities [19, 20, 21, 22, 23].

Among others, boron‐based structures, such as borocarbonitride, borophene, or metal boride, have proven to be promising structures for SCs [24, 25, 26]; however, the use of boron hydride clusters, often called boranes, has been seemingly overlooked. Instead, boranes have been typically used hitherto as substitutes for benzene rings in metal‐organic frameworks [27, 28, 29, 30, 31] and covalent‐organic frameworks [32, 33, 34].

Borane and carborane clusters are a unique class of compounds with unusual 3D polyhedral geometry. They comprise triangular three‐centered two‐electron bonds that result in the delocalization of electrons within their clusters, and hence, they are generally electron deficient in nature. Most commonly used clusters are structurally derived from the icosahedral [*closo‐*B_12_H_12_]^2−^ either by vertex abstraction to give, e.g., [*nido*‐B_11_H_14_]^−^ and *nido*‐B_10_H_14_, or vertex substitution with a nonboron atom to give, e.g., the carboranes [CB_11_H_12_]^−^ or C_2_B_10_H_12_. The negative charge of anionic (car)borane clusters can be compensated by inorganic or organic cations, most commonly Na^+^, K^+^, and Cs^+^ in the former case and alkyl ammonium ([R_4_N]^+^) and aryl phosphonium ([R_4_P]^+^) in the latter. Heteroatoms can be introduced onto the periphery of the borane cluster also by coordination with two‐electron donor ligands to give, for example, *arachno*‐B_10_H_12_L_2_ or *arachno*‐B_9_H_13_L, where L can be, e.g., SR_2_, R_3_N, pyridines, or acetonitrile. Recently, we developed a new type of porous polymer, named “activated borane” (**ActB**), which is based on borane clusters connected by organic moieties using B—B and B—C bonds. **ActB** is synthesized by simple cothermolysis of *nido‐*boranes such as decaborane(14) (*nido*‐B_10_H_14_) and aliphatic or aromatic small molecules [35, 36, 37]. The focus of this article is the preparation of **ActB** containing nitrogen and the testing of the applicability of the resultant **ActB** materials as SCs.

Despite considerable progress in developing high‐performance SC electrodes, achieving a balance between high capacitance, long‐term cycling stability, and effective charge transport remains a significant challenge, particularly in acidic electrolytes. Incorporating heteroatom doping alongside hierarchical porosity has proven to be an effective approach to address these issues by increasing active sites and improving ion diffusion pathways. Here, we present a straightforward cothermolysis method to synthesize a nitrogen‐doped porous borane cluster network. These materials exhibit excellent electrochemical properties when used as an electrode in acidic SCs. Comprehensive electrochemical studies highlight the combined benefits of nitrogen doping and porous structure in enhancing energy storage performance, offering valuable insights for the design of future electrode materials for advanced energy storage technologies.

## 2 Results and Discussion

### 2.1 Synthesis and Characterization

**SCHEME 1:**
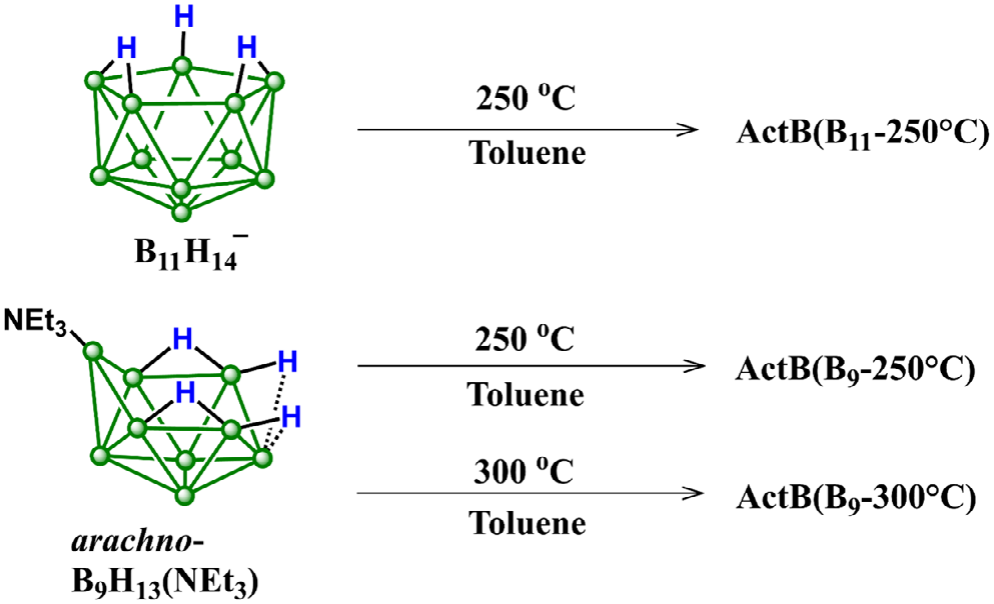
Synthesis of **ActB**s. Green circles represent BH groups.

In order to introduce nitrogen into the structure of **ActB,** we have done the co‐thermolysis using [Et_3_NH][*nido*‐B_11_H_14_] and *arachno*‐B_9_H_13_(NEt_3_); see Scheme 1. In the first case, the anionic borane cluster [*nido*‐B_11_H_14_]^−^ is charge balanced by a triethylammonium ([Et_3_NH]^+^) cation. In the second case, the *arachno*‐B_9_H_13_ cluster is bound through a coordination bond with NEt_3_. In both cases, the cothermolysis with toluene at 250°C for 24 h gave dark powders denoted as **ActB**(B_11_‐250°C) and **ActB**(B_9_‐250°C), respectively. To test if the increased temperature of the reaction has any effect on the resulting properties, we also performed cothermolysis of *arachno*‐B_9_H_13_(NEt_3_) in toluene at 300°C for 24 h, denoted **ActB**(B_9_‐300°C). The reaction was done analogously to previous **ActB** syntheses [34, 35]. In short, in an Ar‐filled glovebox, 400 mg of the cluster was suspended in 5 mL of dry toluene, and the reaction mixture was transferred to a stainless‐steel autoclave heated at a preset temperature for 24 h. The resulting dark solid was Soxhlet extracted with toluene and dried under vacuum; for details, see SI.

**FIGURE 1:**
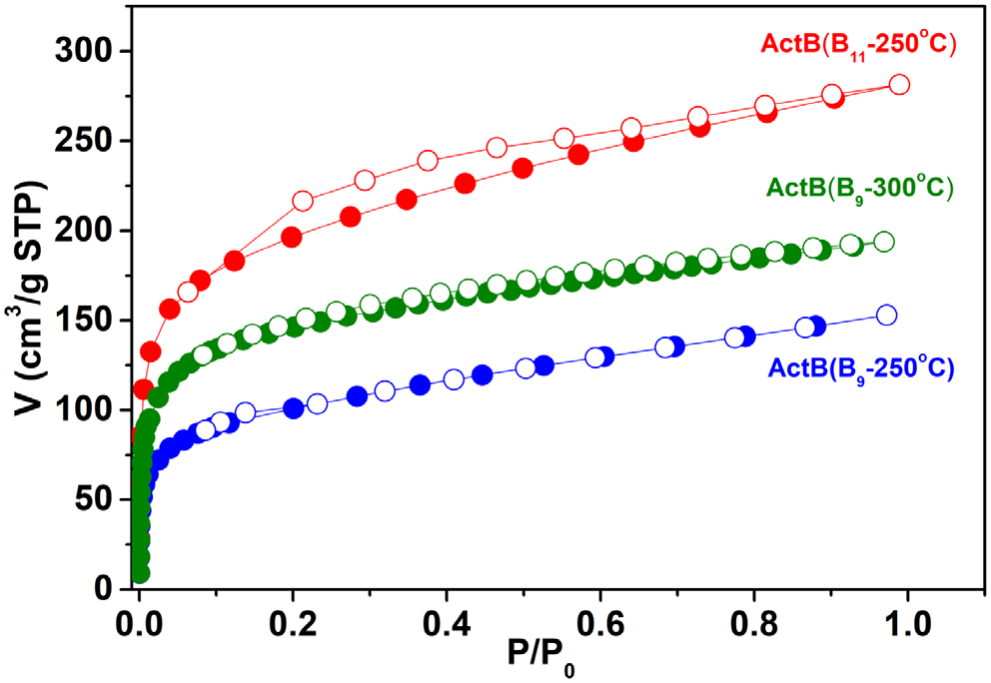
Adsorption isotherms of Ar at 87 K for **ActB**(B_11_‐250°C), **ActB**(B_9_‐250°C), and **ActB**(B_9_‐300°C).

Unfortunately, all **ActB** materials are amorphous, and therefore, exact structure was not possible to obtain. The adsorption isotherms of Ar measured at 87 K (Figure 1, tabular data in tables S1–S3) display a microporous nature of all the **ActB**s, as seen in Figure 1. In the case of **ActB** (B_11_‐250°C), the desorption branch forms a discernible hysteresis, which might suggest the presence of mesoporosity. The specific surface area is highest for **ActB** (B_11_‐250°C), while for both **ActB**s originating from the *arachno*‐B_9_H_13_(NEt_3_) cluster, the specific surface area is lower, as shown in Table 1. For all three samples, the pore size distributions (see Figure S1) display two maxima between 1 and 2 nm, which are in line with **ActB**s prepared from *nido*‐B_10_H_14_ [34]. Elemental analysis (Table 1) of all **ActB**s revealed that the highest content of nitrogen was in the sample **ActB** (B_9_‐250°C) and that with increasing temperature of synthesis, the content of nitrogen decreased. Since the electrochemical properties of **ActB** (B_9_‐300°C) were also inferior in comparison with **ActB** (B_9_‐250°C), we did not test cothermolysis at yet higher temperatures.

**TABLE 1:** Specific surface areas, pore diameters, pore volumes, and CHN analysis for **ActB**(B_11_‐250°C), **ActB**(B_9_‐250°C), and **ActB**(B_9_‐300°C).

Sample	*S*_BET_, m^2^g^−1^ a	Pore size, nm^b^	*V* pore, cm^3^g^−1^ c	C, %	H, %	N, %
**ActB**(B_11_‐250°C)	626	0.9, 1.5^d^	0.36	43.81	6.82	2.31
**ActB**(B_9_‐250°C)	360	1.0, 1.6^d^	0.19	42.59	6.07	3.33
**ActB**(B_9_‐300°C)	476	0.9, 1.6^d^	0.25	35.52	4.4	2.15

a BET specific surface area.

b pore size maxima calculated by the MDFT method.

c Total pore volume at p/p0 = 0.99.

d two pore size maxima were found.

**FIGURE 2:**
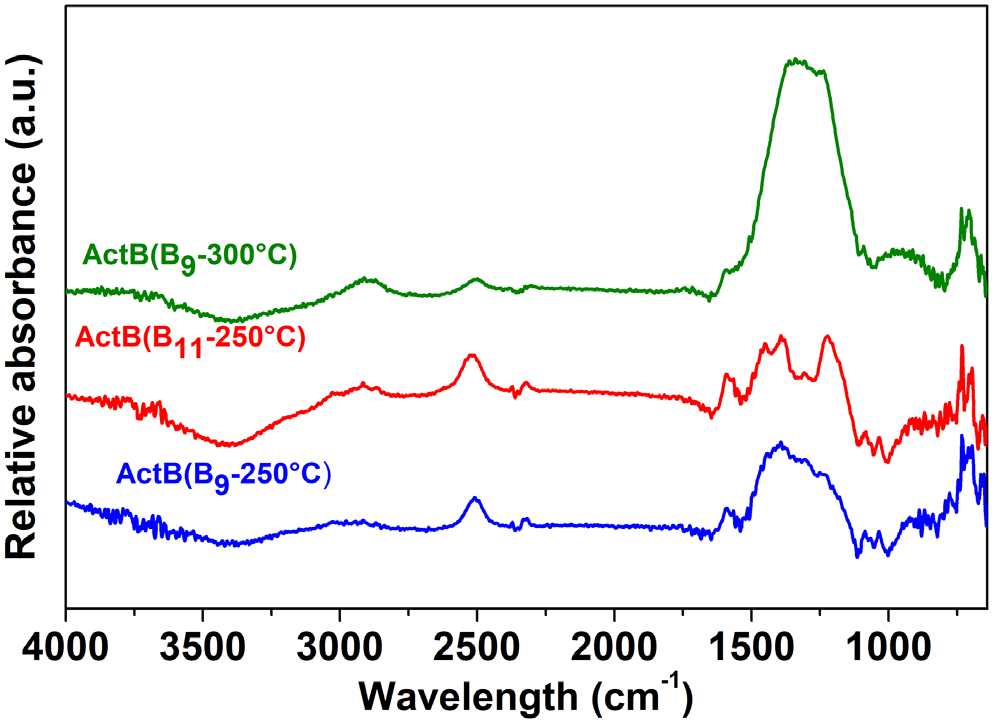
FTIR for **ActB**(B_9_‐250°C) (blue), **ActB**(B_11_‐250°C) (red), and **ActB**(B_9_‐300°C) (green).

**FIGURE 3:**
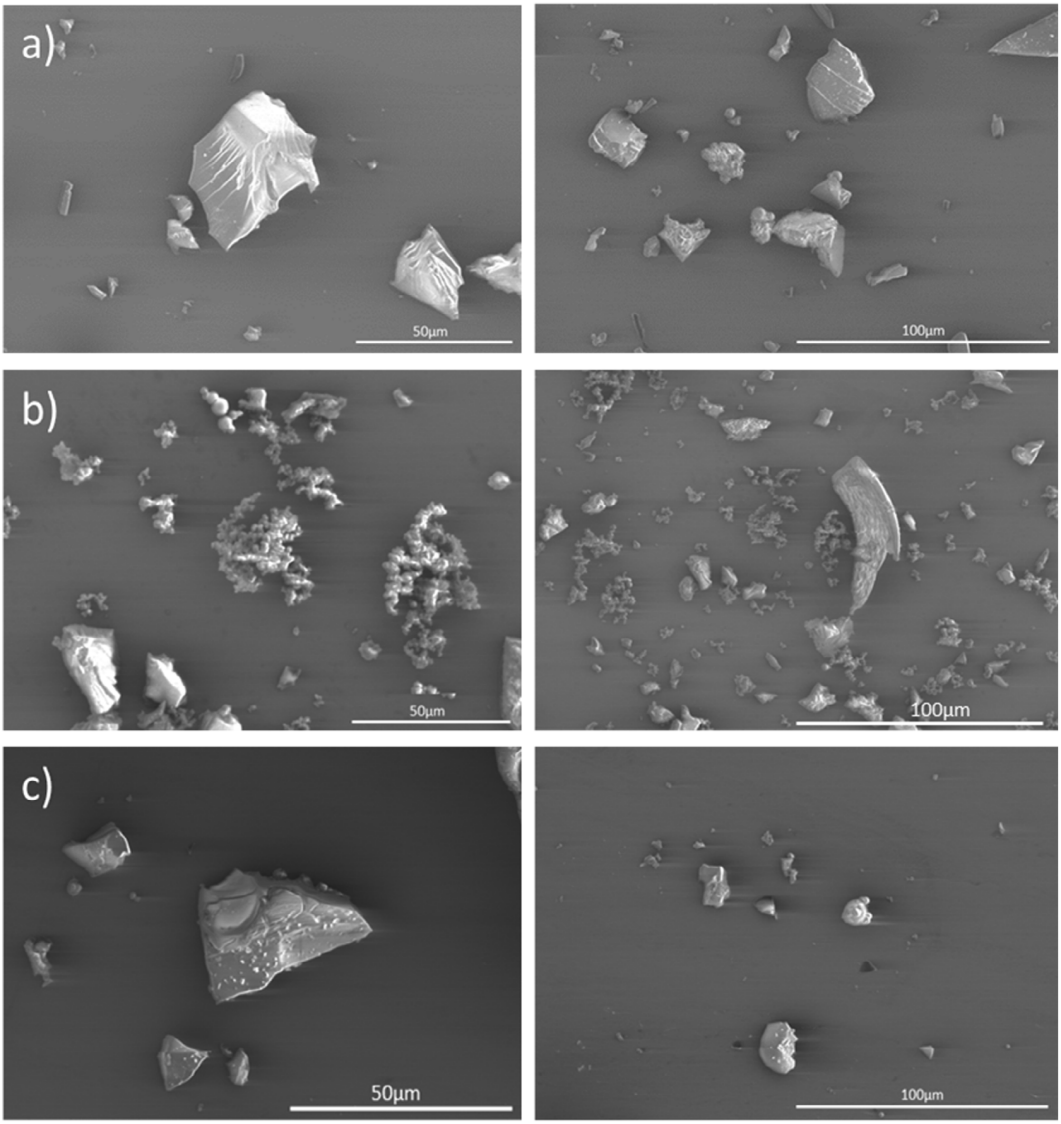
SEM images of (a) **ActB**(B_11_‐250°C) top, (b) **ActB**(B_9_‐250°C) middle, and (c) **ActB**(B_9_‐300°C) bottom.

The fourier transform infrared spectroscopy (FTIR) spectra (Figure 2) of all samples display expected vibrations of B—H bonds around 2500 cm^−1^ and intensive skeletal vibrations below 1500 cm^−1^. Similarly to other **ActB**s published earlier, we did not observe “bridging” *μ*‐hydrogens (B—H—B) around 1900 cm^−1^ present in *nido*‐ and *arachno*‐boranes, implying that during the cothermolysis, the open borane clusters lose their bridging hydrogen atoms as dihydrogen, forming closed polyhedral (*closo‐*) clusters as a result. The scanning electron microscopy (SEM) images for all three samples show irregular shapes of particles with particle sizes ranging from a few microns to tens of microns; see Figure 3.

**FIGURE 4:**
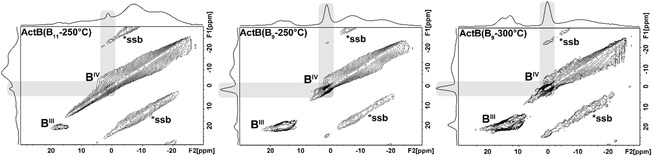
Experimental 2D ^11^B 3Q/MAS NMR spectra of **ActB**(B_11_‐250°C), **ActB**(B_9_‐250°C), and **ActB**(B_9_‐300°C) samples, with gray boxes highlighting B^IV^ coordinated atoms.

In order to obtain insights about the structure of prepared **ActB**s, we have performed a detailed multinuclear NMR study recording ^1^H, ^11^B, ^13^C, and ^15^N solid‐state NMR (ssNMR) spectra. Firstly, 2D ^11^B 3Q/MAS NMR spectra (Figure 4) clearly confirm the amorphous and multicomponent structure of the samples, as reflected by a broad pattern (with several overlapped signals) ranging from −25 to 10 ppm with two maxima at around −15 and −7 ppm that are typical for **ActB** materials already discussed in the literature [36]. Solution‐phase NMR of [Et_3_NH][*nido*‐B_11_H_14_] comprises just three peaks in a region of the ^11^B‐spectrum spanning from −13 to +17 ppm, which is similar to the dominant region of the 2D ^11^B 3Q/MAS NMR spectra of **ActB**(B_11_‐250°C), perhaps suggesting some degree of structural integrity. The solution‐phase NMR of *arachno*‐B_9_H_13_(NEt_3_) [38] spans a greater region of the ^11^B‐spectrum from +16 to −42 ppm, and it is interesting that the 2D ^11^B 3Q/MAS NMR spectra of **ActB**(B_9_‐250°C) and **ActB**(B_9_‐300°C) samples both show downfield chemical shifts at around the +15 ppm in the ^11^B ssNMR spectrum. Again, this could suggest some degree of structural integrity between the **ActB** materials and their molecular starting materials; however, the link is tentative. In addition to these broad patterns, additional sharper and more distinct signals are obvious in the ^11^B spectra of all new **ActB** materials at around 0 ppm (Figure 4, highlighted by gray boxes). We believe these peaks correspond to tricoordinated (B^III^) and tetracoordinated boron (B^IV^) atoms within the **ActB** material matrix. The chemical shift of B^IV^ is at *δ*_iso_ = 1.5 ppm, and the intensity of the peak is proportional to the content of coordinated Et_3_N‐B≤ species, which follows the order **ActB**(B_9_‐300°C) > **ActB**(B_9_‐250°C) > **ActB**(B_11_‐250°C). The peak at *δ*_iso_ = 18.5 ppm with *C*_Q_ = 2.6 MHz and *η *= 0.2 is attributable to B^III^ atoms that were expelled from the borane clusters. We have previously observed the expulsion of boron atoms upon heating of *nido‐*boranes during the synthesis of **ActB**s, as well as during thermal substitution of *nido*‐B_10_H_14_ at 200°C [35, 36, 39]. As expected, thermolysis at higher temperatures and the presence of a more open *arachno* borane cluster (B_9_H_13_NEt_3_) result in the formation of a larger amount of expelled boron species.

**FIGURE 5:**
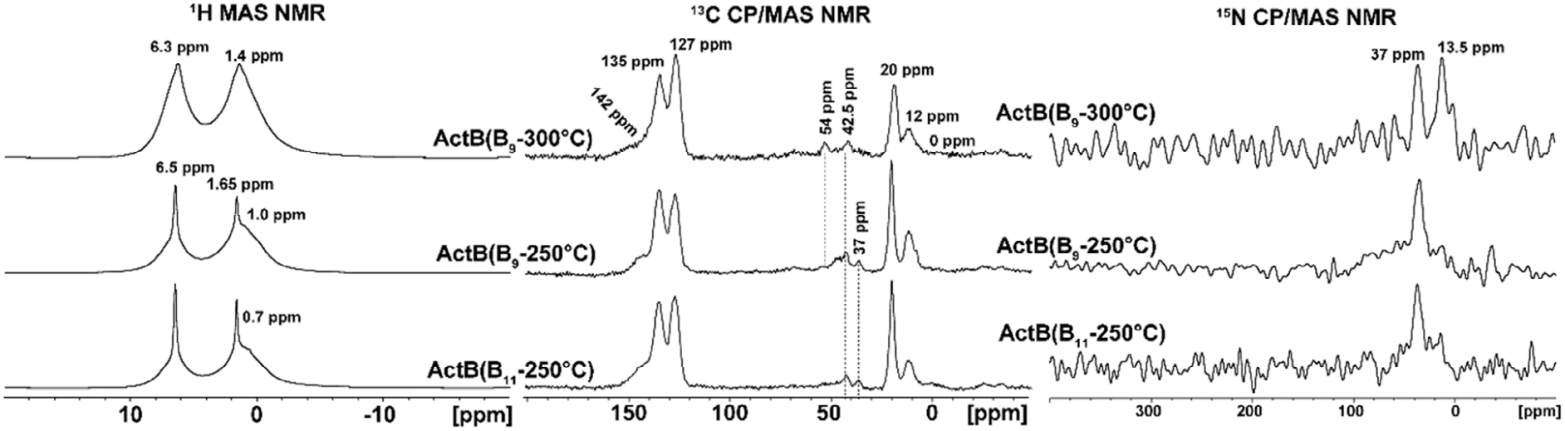
Experimental ^1^H MAS NMR (left‐hand column), ^13^C CP/MAS NMR (middle column), and ^15^N CP/MAS NMR (right‐hand column) spectra of **ActB**(B_11_‐250°C), **ActB**(B_9_‐250°C), and **ActB**(B_9_‐300°C) samples.

All ^13^C CP/MAS NMR spectra of **ActB**s (Figure 5, middle column) exhibit five major peaks ranging from 12 to 142 ppm and minor peaks at 0 , 37 , 42.5 , and 54 ppm. The peak at 12 ppm and peaks ranging from 37  to 54 ppm were assigned to methyl and methylene carbons of NEt_3_H^+^/Et_3_N. The peaks located at 20, 127, 135, and 142 ppm correspond to methyl groups and nonequivalent groups of aromatic carbons from immobilized toluene. The minor peak at 0 ppm was attributed to carbon atoms bonded to the borane cluster forming nodal points, similar to boron carbide systems [40, 41].

The presence of several relatively well‐resolved peaks in the region 37–54 ppm indicates different bonding of [NEt_3_H]^+^/Et_3_N moieties in **ActB**s based on the temperature of synthesis. Attribution of these peaks was accomplished based on ^13^C hr‐NMR data of the starting clusters [Et_3_NH][*nido*‐B_11_H_14_] and *arachno*‐B_9_H_13_(NEt_3_) [38]. **ActB**s prepared at 250°C contain two specific peaks at 37  and 42.5 ppm; the latter can be assigned to the ionic form [Et_3_NH]^+^, whereas **ActB**(B_9_‐300°C) contains peaks at 42.5  and 54 ppm, where the peak at 54 ppm corresponds to the Et_3_N bonded via a coordination bond. The minor signal at 37 ppm is of unknown origin—possibly a signal from CH_3_–N or –CH_2_–N groups [42].

The ionic/coordination bonding character of [NEt_3_H]^+^/Et_3_N was also confirmed by ^15^N CP/MAS NMR spectroscopy; see Figure 5, right column [43, 44, 45]. **ActB**s prepared at 250°C display one sharp signal at 37 ppm and one broad signal in the range of 0–100 ppm attributable to the ionic form [NEt_3_H]^+^. While **ActB**(B_9_‐300°C) displays two relatively sharp signals at 37  and 13.5 ppm, where the signal at 13.5 ppm corresponds to coordinatively bonded Et_3_N.

Finally, the ^1^H MAS NMR spectra of all investigated **ActB**s (Figure 5, left column) reveal three broad signals at 0.7/1.0 ppm, 1.4, and 6.3 ppm, attributable to signals from the methyl group of the [NEt_3_H]^+^/Et_3_N, the methyl group of toluene, and aromatic protons of toluene, respectively. Those peaks are further overlapped with signals from protons of borane clusters [35, 46] and a signal from the methylene group of the [NEt_3_H]^+^/Et_3_N. Moreover, in the cases of **ActB**(B_11_‐250°C) and **ActB**(B_9_‐250°C), the ^1^H MAS NMR spectra indicate a two‐component character of the structure, where the broad peaks correspond to the rigid fraction and the narrow peaks are attributed to relatively mobile parts. In the case of **ActB**(B_9_‐300°C), the ^1^H MAS spectrum indicates only the rigid structure. In all cases, signals from bridging *µ*‐hydrogens of *nido* and *arachno* clusters are not detected, suggesting the transformation of open clusters into the closed clusters (*closo*) and/or full substitution of the *µ*‐hydrogen atoms. This is in line with IR measurement and our previous results on **ActB**s [35, 36].

The ssNMR analysis could be summarized as follows: (a) **ActB**(B_9_‐300°C) is more rigid than **ActB**s prepared at 250°C, probably because of the higher degree of crosslinking, which is in line with the lower content of hydrogen; (b) **ActB**(B_11_‐250°C) contains the highest number of ionically bonded [Et_3_NH]^+^, whereas **ActB**(B_9_‐300°C) contains the most coordinatively bonded Et_3_N; (c) the amount of boron expelled from borane clusters follows the order **ActB**(B_9_‐300°C) > **ActB**(B_9_‐250°C) > **ActB**(B_11_‐250°C); (d) in all cases open borane clusters turned into closed clusters during the synthesis, which is in line with FTIR results.

XPS was used to better understand the surface chemical composition of the novel **ActB** materials. Due to the experimental limitations, the samples were exposed to air during the sample preparation. This resulted in fast adsorption of water molecules, introducing oxygen into the samples.

**FIGURE 6:**
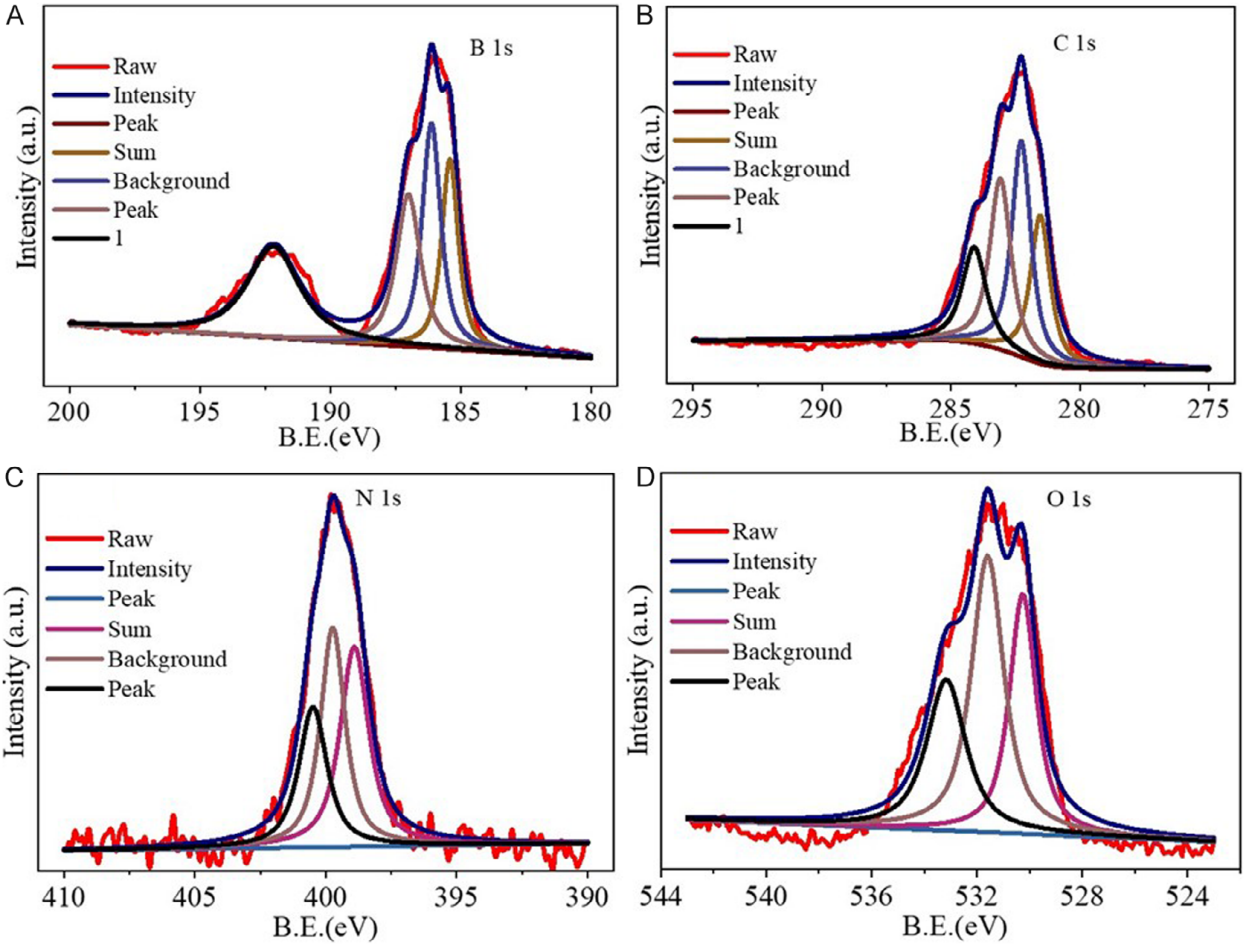
(a) XPS spectra of **ActB**(B_9_‐250°C) for B 1S, (b) C 1s, (c) N 1s, and (d) O 1s.

The complete XPS spectrum of **ActB**(B_9_‐250°C) is presented in Figure 6. The high‐resolution B 1s spectrum (Figure 6a) displays a multicomponent peak that can be deconvoluted into three distinct contributions at binding energies of 185.4, 186.1, and 187.0 eV, corresponding to various B—C bonding environments. In addition, a broad shoulder centered at 192.2 eV is observed, which can be attributed to the overlapping signals of B—O and B—N bonds, indicating partial oxidation and successful nitrogen incorporation [47, 48]. The C 1s region (Figure 6b) reveals four primary peaks at approximately 281.5, 282.3, 283.1, and 284.1 eV. These are assigned to C—B, C—C, C—O, and O—C=O species, respectively, suggesting the coexistence of both sp^2^ carbon and oxygen‐containing functional groups within the carbon framework [8]. In the N 1s spectrum (Figure 6c), a broad envelope comprising three components is identified at 398.5, 399.7, and 400.5 eV. These are associated with B—N, C=N, and tetraalkylammonium‐type nitrogen species, respectively, confirming the successful doping of nitrogen in different chemical environments [47, 49]. The O 1s spectrum (Figure 6d) exhibits well‐defined peaks at 530.3, 531.5, and 533.2 eV, corresponding to C=O, O—C=O, and B—O bonds, respectively. The presence of these oxygen functionalities suggests partial surface oxidation during the post‐treatment [47, 50]. Those results show that the boron and nitrogen atoms are integrated into the carbon network and are in line with other characterization techniques.

### 2.2 Electrochemical Performance Evaluation of ActBs in Various Electrolytes

**FIGURE 7:**
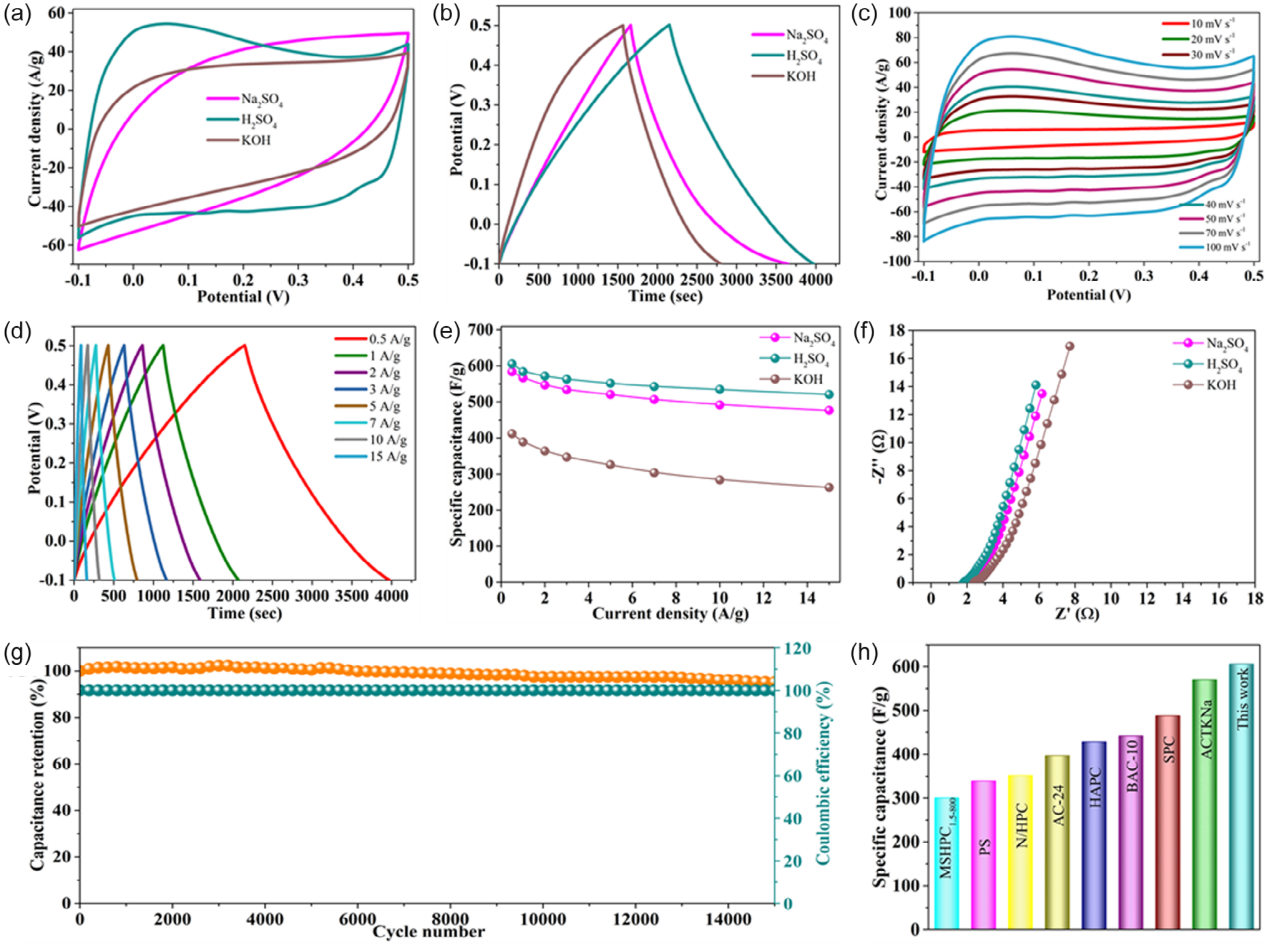
(a) CV curves and (b) GCD curves of different electrolytes using **ActB**(B_9_‐250°C. (c) CV curves at the scan rates and (d) GCD curves at different current densities using 0.5 M H_2_SO_4_, (e) specific capacitance with different electrolytes, (f) EIS Nyquist plots, (g) cycling performance at 10 A g^−1^, and (h) bar chart comparison of the specific capacitance of **ActB**(B_9_‐250°C) with previously reported values from the literature.

The electrochemical behavior of **ActB**(B_9_‐250°C) was systematically investigated using a three‐electrode setup in different aqueous electrolytes, including 0.5 M H_2_SO_4_, 1.0 M Na_2_SO_4_, and 1.0 M KOH (Figures 7 and S7). Cyclic voltammetry (CV) and galvanostatic charge–discharge (GCD) measurements reveal excellent capacitive performance and stability, particularly in acidic media. As illustrated in Figure 7a, the CV curves at a scan rate of 50 mV  s^−1^ display quasirectangular shapes, suggesting that the charge storage mechanism is dominated by electric double‐layer capacitance (EDLC), with capacitive behavior predominantly governed by ion adsorption/desorption at the electrode–electrolyte interface. Notably, deviation from ideal rectangularity implies pseudocapacitive contributions, likely arising from surface‐bound oxygen‐containing and nitrogen‐functional groups. These functional groups contribute significantly to the redox behavior of the material, as evidenced by the CV profiles presented in Figure 7c. The CV curves recorded at various scan rates (10–100 mV s^−1^) exhibit well‐retained shapes with minimal distortion, indicating excellent rate capability and efficient ion transport within the electrode. Notably, similar CV characteristics were observed across all tested samples, **ActB**(B_9_‐250°C), **ActB**(B_9_‐300°C), and **ActB**(B_11_‐250°C) (Figures S8 and S9), highlighting their strong electrochemical reversibility and stable capacitive behavior under dynamic charge–discharge conditions. The GCD curves shown in Figures 7b, d display nearly symmetric, isosceles triangular profiles across a wide range of current densities (0.5–15 A g^−1^), indicating excellent charge/discharge reversibility and capacitive efficiency. Among the tested materials, **ActB**(B_9_‐250°C) demonstrated the longest discharge time, which is consistent with its superior specific capacitance, as illustrated in Figure 7e. Furthermore, comparable GCD behavior was observed for **ActB**(B_9_‐250°C), **ActB**(B_9_‐300°C), and **ActB**(B_11_‐250°C) (Figures S8 and S9), underscoring their stable electrochemical response and robust capacitive performance under varying current densities. Notably, **ActB**(B_9_‐250°C) exhibited the highest specific capacitance of 607 F g^−1^ at a current density of 0.5 A g^−1^ when tested in 0.5 M H_2_SO_4_, outperforming its performance in Na_2_SO_4_ (583 F g^−1^) and KOH (410 F g^−1^) under identical conditions [14, 51]. The enhanced capacitive behavior in the acidic electrolyte is likely attributed to improved ionic conductivity, efficient proton transport, and favorable electrode–electrolyte interactions that facilitate faster redox kinetics. The difference in capacitance between H_2_SO_4_ and KOH indeed reflects the change in the dominant charge‐storage mechanism under different pH conditions. Because borane clusters are intrinsically more stable in acidic media, H_2_SO_4_ was chosen as the electrolyte of choice. To fully assess pH effects, we also performed measurements in KOH. Although the capacitance is lower in alkaline solution, the electrodes still showed good stability and reversible behavior. In an acidic electrolyte, the high proton availability enables fast proton‐coupled electron‐transfer reactions at nitrogen and boron sites, which contribute strongly to Faradaic pseudocapacitance and result in the higher value of 607 F g^−1^. In alkaline media, these proton‐driven redox processes are largely suppressed, so the charge storage is dominated by electric double‐layer formation, giving a lower capacitance (410 F g^−1^). Importantly, SEM–EDX analysis and electrochemical cycling confirm that the structural integrity of the **ActB** materials is maintained.

Electrochemical impedance spectroscopy (EIS) analysis (Figure 7f) reveals a low charge transfer resistance (*R*_ct_), as evidenced by the smaller semicircle diameter in the Nyquist plot for **ActB**(B_9_‐250°C), compared to **ActB**(B_9_‐300°C) and **ActB**(B_11_‐250°C) (Figure S10). This implies improved electrical conductivity and faster electron transport within the electrode, further explaining its superior capacitive behavior. Long‐term cycling stability was assessed by CV testing at 10 mV s^−1^ over 15,000 cycles (Figure 7g). The electrode retained 95% of its initial capacitance, with a Coulombic efficiency close to 100%, indicating robust cycling stability and excellent electrochemical reversibility. Finally, a comparative analysis (Figure 7h) with previously reported carbon‐based and hybrid materials, such as sulfur‐doped porous carbon, nitrogen‐doped hierarchical porous carbon (NHPC), and hemp‐derived activated carbon (AC) (HAC), demonstrates the competitive advantage of **ActB**(B_9_‐250°C). It not only offers higher specific capacitance but also exhibits outstanding stability, making it a promising candidate for high‐performance energy storage applications. The remarkable electrochemical performance of **ActB**(B_9_‐250°C), despite its relatively lower surface area, is likely attributed to the synergistic effects of high nitrogen content and the nature of nitrogen bonding within the framework. To examine how acidic conditions influence the porosity of the **ActB** materials, we treated all three samples in 0.5 M H_2_SO_4_, the electrolyte in which the best electrochemical performance was obtained. After treatment, nitrogen sorption measurements showed a clear drop in porosity. SEM images confirmed that the overall morphology remained unchanged, while EDX revealed a substantial content of sulfur (Figures S4–S6, Table S7). This strongly suggests that sulfate species adsorb on the surface and partially block the pores, explaining the observed decrease in accessible specific surface area. Although **ActB**(B_11_‐250°C) initially has a higher specific surface area than **ActB**(B_9_‐250°C), its electrochemical performance is noticeably lower. This indicates that surface area is not the dominant factor here. As shown by solid‐state NMR and XPS, **ActB**(B_11_‐250°C) contains a larger fraction of ionic nitrogen [Et_3_NH]^+^, which improves wettability but provides weak electronic coupling within the borane network. In contrast, **ActB**(B_9_‐250°C) has a more favorable balance of ionic and covalently bound Et_3_N‐type nitrogen. Covalent nitrogen promotes partial B–N hybridization and better charge delocalization, leading to improved electronic transport, while the ionic sites still support efficient ion diffusion. This combination, collected with the moderate and more accessible porosity of **ActB**(B_9_‐250°C), results in its superior capacitance.

### 2.3 Asymmetric Supercapacitor Based on ActB(B_9_‐250°C)//AC

**FIGURE 8:**
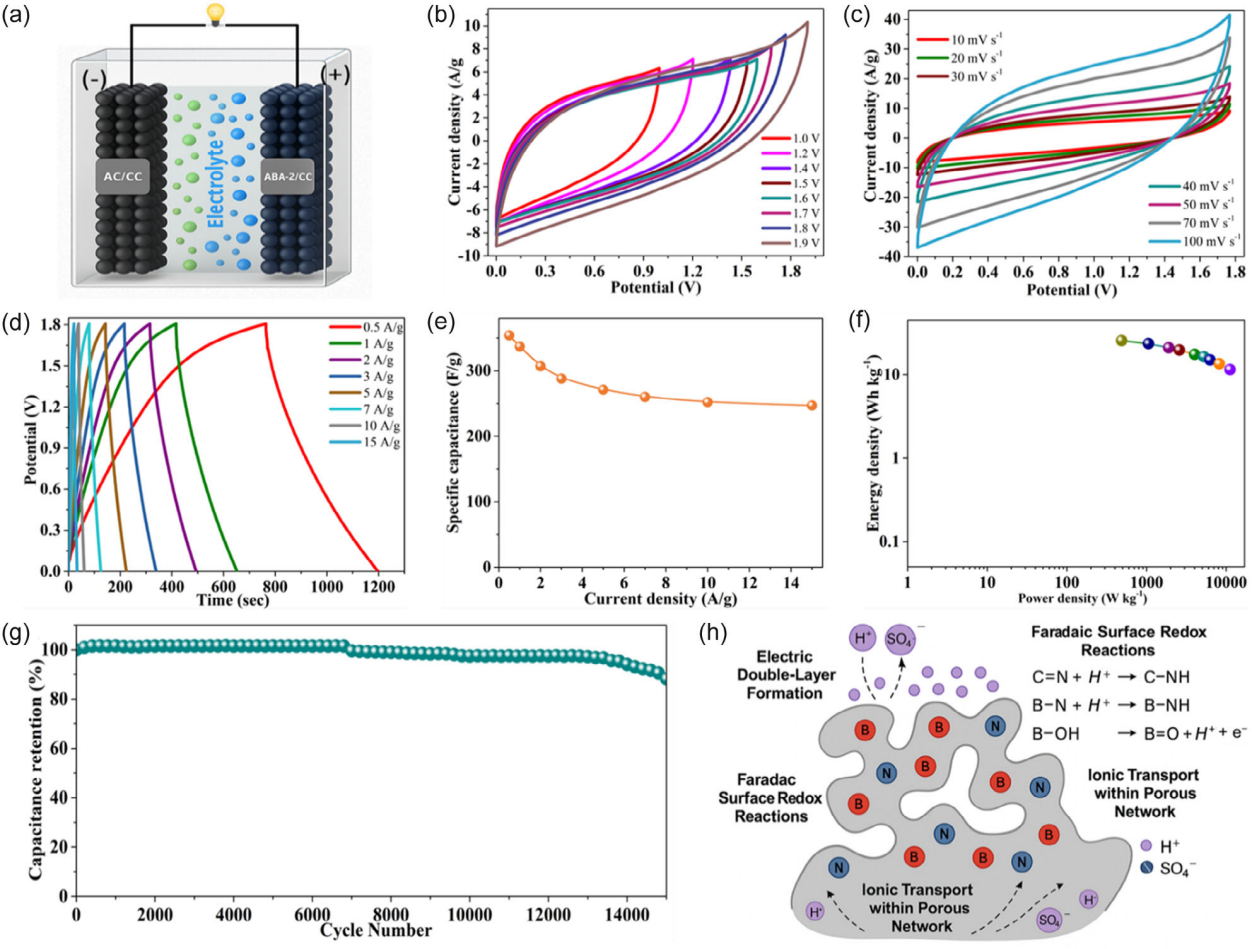
(a) Schematic illustration of the **ActB**(B_9_‐250°C)//AC ASC device, (b) CV curves at different potentials (1.0–1.9 V) at a scanning rate of 10 mV s^−1^, (c) CV curves at different scan rates, (d) GCD curves at different current densities, (e) specific capacitance, (f) Ragone plots, and (g) cycling stability test at 10 A g^−1^. (h) Energy storage mechanism in borane‐based electrode.

Following the promising electrochemical results obtained in a three‐electrode configuration, an asymmetric supercapacitor (ASC) was fabricated using **ActB**(B_9_‐250°C) as the positive electrode and AC as the negative electrode, as illustrated in Figure 8a. To ensure optimal device performance, the mass balance between the electrodes and their individual potential windows was precisely optimized through CV and GCD analyses in a three‐electrode system. The AC electrode exhibited a stable operating range from −1.0 to 0 V (Figure 8b), which is typical of EDLCs. The **ActB**(B_9_‐250°C) electrode demonstrated pronounced pseudocapacitive behavior with redox reactions contributing to charge storage, thereby allowing a broader working voltage window. The synergistic combination of these two electrodes facilitated an extended cell voltage of up to 1.8 V, a significant improvement over traditional symmetric systems.

A PVA–H_2_SO_4_ gel electrolyte was employed in the ASC device, providing multiple advantages such as enhanced ionic conductivity, improved safety, and the possibility of flexible, solid‐state device integration. The electrochemical reversibility and rate capability of the ASC were investigated through CV measurements at various scan rates (10–100 mV s^−1^), as shown in Figure 8c. The nearly rectangular and symmetric shapes of the CV profiles at increasing scan rates confirmed the excellent rate capability and minimal resistive losses within the device. GCD measurements (Figure 8d) further confirmed the pseudocapacitive behavior of the **ActB**(B_9_‐250°C) electrode, as evidenced by the nonlinear discharge profiles. The curved shape indicates the presence of Faradaic redox reactions, facilitated by the nitrogen‐rich borane framework that provides multiple redox‐active sites. Minimal iR drop across current densities suggests good electrical conductivity and rapid ion transport within the electrode. The gradual decrease in specific capacitance with increasing current density reflects diffusion‐limited kinetics, yet the high capacitance retention at 15 A g^−1^ underscores the material's excellent rate performance and electrochemical robustness.

As expected, the specific capacitance decreased with increasing current density (Figure 8), ranging from 354 F g^−1^ at 0.5 A g^−1^ to 247 F g^−1^ at 15 A g^−1^, due to ion diffusion limitations at higher rates. The high capacitance at lower current densities reflects effective Faradaic charge storage, facilitated by the nitrogen‐rich borane framework offering abundant redox‐active sites. Even at elevated current densities, the material retains significant capacitance, indicating good electrical conductivity, fast ion transport, and structural stability. This highlights the strong electrode–electrolyte interaction and efficient charge transfer kinetics within the **ActB**(B_9_‐250°C) electrode. The energy and power densities were analyzed via a Ragone plot (Figure 8f), with the device delivering a high energy density of 25.6 Wh kg^−1^ at a power density of 486.2 W kg^−1^. Notably, the ASC exhibited a wide operational range, with minimal energy loss at higher power densities, surpassing the performance of many previously reported carbon‐ and polymer‐based SC systems (see Table S8). Furthermore, the cycling stability of the ASC device was evaluated over 15,000 charge–discharge cycles at a high current density of 10 A g^−1^. As depicted in Figure 8g, the device retained approximately 88% of its initial capacitance, confirming its excellent electrochemical durability and mechanical stability during prolonged operation.

The proposed energy storage mechanism of the nitrogen‐doped borane cluster framework, as illustrated in the schematic, operates through a synergistic combination of EDLC and Faradaic surface redox reactions (Figure 8h). In acidic electrolyte, protons (H^+^) and sulfate ions (SO_4_^2−^) interact with the porous, heteroatom‐doped electrode surface. The high surface area facilitates the formation of an electric double layer via the reversible physical adsorption of ions, contributing to non‐Faradaic charge storage. Concurrently, redox‐active sites introduced by nitrogen and boron functionalities enable Faradaic reactions such as the protonation of C=N and B–N groups, forming C–NH and B–NH species, respectively. Additionally, the presence of B–OH groups allows for redox cycling with reversible conversion to B=O. These reactions enhance the pseudocapacitive behavior of the electrode. The interconnected porous architecture ensures efficient ionic transport throughout the material, minimizing diffusion resistance and enabling high‐rate charge–discharge performance. This dual mechanism underscores the material's potential as a high‐performance electrode for next‐generation SCs. Finally, the protonated [Et_3_NH]^+^ sites primarily enhance ionic transport and interfacial wettability by establishing a dynamic hydrogen‐bonding network at the electrode–electrolyte interface, which accelerates proton/ion diffusion and charge recovery during redox events. These ionic species also modulate the local electric field, improving accessibility of electrochemically active boron sites, an effect that is especially important under acidic and neutral pH, where proton conduction dominates. In contrast, covalently bound Et_3_N functions as an electron‐donating dopant: the nitrogen lone pair interacts with electron‐deficient boron clusters, promoting local charge delocalization and partial B–N hybridization, increasing the electronic density of states near the Fermi level and thereby facilitating electron transport and pseudocapacitive storage. Covalent N‐doping further stabilizes the borane framework by mitigating electron depletion during repeated redox cycling, improving long‐term durability. The superior performance of **ActB**(B_9_‐250°C) is attributed to an optimal ionic:covalent nitrogen ratio at that treatment temperature, which balances fast ion diffusion (ionic N) with high electronic conductivity and structural stability (covalent N).

## 3 Conclusions

In this study, we report the successful synthesis of nitrogen‐doped porous borane cluster‐based networks via a controlled thermolysis strategy. Among the synthesized materials, **ActB**(B_9_‐250°C), prepared by the cothermolysis of *arachno*‐B_9_H_13_(NEt_3_) and toluene at 250°C, exhibited outstanding electrochemical properties. In a three‐electrode configuration, **ActB**(B_9_‐250°C) delivered a remarkable specific capacitance of 607 F g^−1^ at a current density of 0.5 A g^−1^, along with an impressive capacitance retention of 95% after 15,000 charge–discharge cycles, underscoring its long‐term electrochemical stability. An ASC device using AC as the negative electrode, in the **ActB**(B_9_‐250°C)//AC configuration, exhibited excellent energy storage performance, delivering a specific capacitance of 354 F g^−1^ at 0.5 A g^−1^, an energy density of 25.6 Wh kg^−1^, and a power density of 486.2 W kg^−1^, while retaining 88% of its capacitance after 15,000 cycles, indicating outstanding durability. We attribute the superior performance to the synergistic combination of nitrogen doping and the intrinsic characteristics of the borane cluster network, which contribute to enhanced electronic conductivity, abundant redox‐active sites, and efficient ion diffusion pathways. This work highlights the great promise of nitrogen‐doped borane cluster‐based polymers as high‐performance electrode materials for next‐generation energy storage devices, particularly in ASC systems.

## Supporting Information

Additional supporting information can be found online in the Supporting Information section. The Supporting Information containing full experimental details of syntheses, electrochemical experiments, additional characterization data for the **ActB** materials, and instrumental details is available free of charge at www. The authors have cited additional references within the Supporting Information [52, 53, 54, 55, 56, 57, 58, 59, 60, 61, 62, 63, 64, 65, 66, 67, 68, 69]. Additional supporting information can be found online in the Supporting Information section. **Supporting Fig. S1:** Pore size distribution for parent **ActB**s. **Supporting Fig. S2:** Adsorption isotherms of Ar (87 K) after treatment with 0.5 M H_2_SO_4_. **Supporting Fig. S3:** SEM images of **ActB**(B_11_‐250**°**C) (top), **ActB**(B_9_‐250**°**C) (middle), and **ActB**(B_9_‐300**°**C) (bottom). In all cases in the left column is parent **ActB** and in the right column after 0.5 M H_2_SO_4_ treatment. **Supporting Fig. S4:** EDX of **ActB**(B_11_‐250**°**C) after treatment with 0.5 M H_2_SO_4_. **Supporting Fig. S5:** EDX of **ActB**(B_9_‐250**°**C) after treatment with 0.5 M H_2_SO_4_. **Supporting Fig. S6:** EDX of **ActB**(B_9_‐300**°**C) after treatment with 0.5 M H_2_SO_4_. **Supporting Fig. S7:** CV and GCD curves: (a−b) 1.0 M Na_2_SO_4_, and (c−d) 1.0 M KOH using **ActB**(B_9_‐250°C). **Supporting Fig. S8:** (a) CV curves and (b) GCD curves for **ActB**(B_11_‐250°C). **Supporting Fig. S9:** (a) CV curves and (b) GCD curves for **ActB**(B_9_‐300°C). **Supporting Fig. S10:** EIS Nyquist plots for **ActB**(B_9_‐250°C), **ActB**(B_11_‐250°C), and **ActB**(B_9_‐300°C). **Supporting Table S1:** Tabular data for the Ar adsorption isotherm of **ActB**(B_11_‐250°C). **Supporting Table S2:** Tabular data for the Ar adsorption isotherm of **ActB**(B_9_‐250°C). **Supporting Table S3:** Tabular data for the Ar adsorption isotherm of **ActB**(B_11_‐300°C). **Supporting Table S4:** Tabular data for the Ar adsorption isotherm of **ActB**(B_11_‐250°C) after treatment with 0.5 M H_2_SO_4_. **Supporting Table S5:** Tabular data for the Ar adsorption isotherm of **ActB**(B_9_‐250°C) after treatment with 0.5 M H_2_SO_4_. **Supporting Table S6:** Tabular data for the Ar adsorption isotherm of **ActB**(B_9_‐300°C) after treatment with 0.5 M H_2_SO_4_. **Supporting Table S7:** Specific surface areas calculated from Ar adsorption at 87 K and nitrogen and sulfur content determined by EDX for **ActB**(B_11_‐250°C), **ActB**(B_9_‐250°C), and **ActB**(B_9_‐300°C) all after treatment in 0.5 M H_2_SO_4_ for 1 h at RT. **Supporting Table S8:** Comparison of electrochemical SC performance with previously obtained results.

## Conflicts of Interest

The authors declare no conflicts of interest.

## Supporting information

Supplementary Material

## Data Availability

The data that support the findings of this study are available from the corresponding author upon reasonable request.
