# Supplementary material for: Nitrogen‐Doped Borane Cluster Network for High‐Performance Supercapacitors Under Universal pH Conditions
Source: ChemSusChem. 2026 Jan 29;19(3):e202502009. doi: 10.1002/cssc.202502009 (PMC12854923; doi:10.1002/cssc.202502009)
Supplement: Supplementary file 1 — Supplementary Material [file CSSC-19-e202502009-s001.pdf]

Supporting Information

Nitrogen-Doped Borane Cluster Network for High-Performance Supercapacitors under Universal pH Conditions

Abhishek Udnoor,[a #] *Samikannu Prabu,*[b#] *Madhan Vinu,*[b#] *Matouš Kloda,*[a] *Andrii Mahun,*[c] *Libor Kobera,*[c] *Michael G. S. Londesborough,*[a] *Kung-Yuh Chiang,*[b*] *Jan Demel*[a *]

[a] Institute of Inorganic Chemistry of the Czech Academy of Sciences, Husinec-Řež 1001, 250 68, Řež, Czech Republic; E-mail: demel@iic.cas.cz

[b] Graduate Institute of Environmental Engineering, National Central University, Tao-Yuan City 32001, Taiwan; E-mail: kychiang@ncu.edu.tw

[c] Institute of Macromolecular Chemistry of the Czech Academy of Sciences, Heyrovského nám. 2, 162 00, Prague 6, Czech Republic

# equal contribution

General Information for Experiments

Materials. $[Et_3NH][nido\text{-}B_{11}H_{14}]$ and *nido*-$B_{10}H_{14}$ (Katchem, Czech Republic) were used as received, *arachno*-$B_9H_{13}(NEt_3)$ was prepared using a standard published procedure [1,2], based on the following three reactions starting with commercially available *nido*-$B_{10}H_{14}$:

$$nido\text{-}B_{10}H_{14} + 2\ Me_2S \longrightarrow arachno\text{-}6,9\text{-}(Me_2S)_2B_{10}H_{12} + H_2 \quad (1)$$

$$arachno\text{-}6,9\text{-}(Me_2S)_2B_{10}H_{12} + 3MeOH \longrightarrow arachno\text{-}B_9H_{13}(Me_2S) + B(OMe)_3 + H_2 \quad (2)$$

$$arachno\text{-}B_9H_{13}(Me_2S) + Et_3N \longrightarrow arachno\text{-}B_9H_{13}(Et_3N) + Me_2S \quad (3)$$

Toluene, *n*-hexane, and cyclohexane (all Lach:Ner, Czech Republic) were dried over Na/benzophenone and freshly distilled before use. The syntheses of **ActB**s were done under Ar using standard Schlenk technique and Ar-filled glove box (PureLab, Inert corp.). Polyvinylidene difluoride (PVDF, average Mw ~534000 by GPC, powder), *N*-methyl-2-pyrrolidone (NMP, C_5H_9NO, anhydrous, 99.5%), and potassium hydroxide (KOH, ACS reagent, ≥85%, pellets, all Sigma Aldrich) were used as received.

Instrumental methods.

SEM images were recorded using a HR-SEM FEI NanoSEM 450 equipped with a circular backscatter and EDX detector, operating at an accelerating voltage of 5 kV. The samples were deposited onto a silicon wafer chip. Adsorption isotherms of Ar at 87 K were recorded using a 3P micro 300 Instrument (3P Instruments) using a CryoTune 87 K. Prior to adsorption experiments, the samples were evacuated at 100 °C for at least 24 h under dynamic vacuum of turbopump. FT-IR spectra were recorded on a Nicolet Avatar spectrometer using an ATR technique with a Si crystal on samples enclosed in a cuvette under Ar atmosphere.

Solid-state ^{1}H, ^{11}B, and ^{15}N NMR spectra were recorded at 16.4 T using a Bruker Avance Neo spectrometer. A 3.2 mm cross-polarization magic angle spinning (CP/MAS) probe was used for the corresponding NMR experiments carried out at Larmor frequencies of $\nu(^{1}H)$ = 700.13 MHz, $\nu(^{11}B)$ = 224.63 MHz, and $\nu(^{15}N)$ = 70.96 MHz, respectively. Solid-state ^{13}C NMR spectra were acquired at 11.7 T using a Bruker AVANCE III HD spectrometer equipped with a 3.2 mm CP/MAS probe operating at Larmor frequency of $\nu(^{13}C)$ = 125.76 MHz. The ^{1}H, ^{11}B, and ^{13}C ssNMR spectra were recorded at the MAS rotation rate of 20 kHz. The ^{15}N ssNMR spectra at 10 kHz spinning speed. The ^{1}H NMR chemical shift was calibrated using adamantane (^{1}H: 1.85ppm), the ^{11}B NMR spectra were referenced to the H_3BO_3 in D_2O (^{11}B: 19.5 ppm), the ^{13}C NMR and the ^{15}N NMR chemical shifts were referenced to solid α-glycine (^{13}C: 176.03 ppm, carbonyl signal and ^{15}N: 34.35 ppm, respectively), as external standards.

The ^{1}H MAS experiments were performed using a 2.27 μs 90˚ pulse with 64 scans and 2 s recycle delay. The ^{11}B 3Q/MAS NMR spectra were acquired with spectral width in both frequency dimensions of 62.5 kHz [3]. The indirect detection period t_1 consisted of 96 increments, each made of 512 scans. The ^{13}C CP/MAS NMR experiments were carried out using a {^{1}H} 90˚ pulse with a length of 2.27 μs, 1.5 ms cross-polarization contact time with 4096 scans and 2 s recycle delay. In the case of ^{15}N CP/MAS NMR experiments, a 7 ms cross-polarization contact time was used with a 4 s recycle delay and ca. 30k scans. The SPINAL-64 decoupling sequence was used in ^{15}N and ^{11}B NMR experiments, and rCW^{ApA} sequence was used in ^{13}C NMR experiments in order to remove heteronuclear interactions [4,5]. All the samples were packed into ZrO_2 rotors and subsequently kept under an inert atmosphere. All experiments were

performed at 298 K. Bruker TopSpin 3.2 pl5 software package was used for the processing of the spectra.

X-ray photoelectron spectroscopy (XPS) analysis was performed on an Rigaku NANO-Viewer X-ray photoelectron spectrometer with an achromatized Al Kα source (15 kV, 150 W). The Ar sputtering experiments were conducted in a circumstantial vacuum environment of 5×10^{-6} Pa and a sputtering acceleration voltage of 3 kV.

Synthesis of Activated Boranes

Synthesis of ActB(B_9-250°C)

400 mg of $B_9H_{13}(Et_3N)$ was suspended in 5 mL of dry toluene, transferred to a stainless-steel autoclave (Berghof BR-300) and heated to 250 °C for 24 h. The obtained black material was washed by Soxhlet extraction with toluene for 24 h under Ar. The resulting black solid was dried in vacuum at 100 °C for 4 h.

Synthesis of ActB(B_{11}-250°C)

400 mg of $[Et_3NH][B_{11}H_{14}]$ was suspended in 5 mL of dry toluene, transferred to a stainless-steel autoclave (Berghof BR-300) and heated to 250 °C for 24 h. The obtained dark material was washed by Soxhlet extraction with toluene for 24 h under Ar. The resulting brown solid was dried in vacuum at 100 °C for 4 h.

Synthesis of ActB(B_9-300°C)

1 g of $B_9H_{13}(Et_3N)$ was suspended in 13 mL of dry toluene, transferred to a stainless-steel autoclave (Berghof BR-300) and heated to 300 °C for 24 h. The obtained dark material was washed by Soxhlet extraction with toluene for 24 h under Ar. The resulting black solid was dried in vacuum at 100 °C for 4 h.

Treatment with H_2SO_4

To understand the processes going on in acidic electrolyte, all three **ActB**s were treated with 0.5 M H_2SO_4 at room temperature for 1 h. After that time the samples were thoroughly washed with H_2O and dried in vacuum. Those samples were then subject of characterization by adsorption of Ar at 87 K, SEM and EDX analyses, we also performed CHN analysis of those samples.

Adsorption isotherms of Ar

Table S1. Tabular data for the Ar adsorption isotherm of **ActB**(B_{11}-250˚C)

Adsorption					
P/P_0	V_{ads} (cm^3/g STP)	P/P_0	V_{ads} (cm^3/g STP)	P/P_0	V_{ads} (cm^3/g STP)
2.56E-05	4.5425	0.005574	79.81573	0.363547	157.5397
4.17E-05	9.06301	0.006892	83.86394	0.401739	160.6425
7.54E-05	13.60313	0.008472	87.81364	0.441798	163.7112
0.00012	18.1383	0.010898	91.44437	0.480592	166.0879
0.000153	22.66156	0.016927	98.05799	0.519889	168.5944
0.000216	27.17928	0.023935	104.3009	0.557298	170.9493
0.000313	31.69783	0.03275	109.8691	0.594787	173.2432
0.000416	36.20637	0.043573	114.5525	0.631834	175.3963
0.000544	40.70015	0.056525	118.5774	0.677375	177.7271
0.000771	45.16333	0.070059	122.342	0.713379	179.6777
0.001064	49.61416	0.084577	125.4868	0.758799	182.0951
0.001394	54.02344	0.100681	128.3024	0.795204	183.4055
0.001774	58.43243	0.136664	133.227	0.843396	185.4852
0.002252	62.81696	0.174916	137.4898	0.890483	187.5624
0.002873	67.14227	0.214052	141.3832	0.936707	189.6083
0.003618	71.42396	0.248248	146.4058	0.982068	191.9141
0.004514	75.66704	0.286139	150.4423		
Desorption					

P/P_0	V_{ads} (cm^3/g STP)	P/P_0	V_{ads} (cm^3/g STP)	P/P_0	V_{ads} (cm^3/g STP)
0.982068	191.9141	0.612457	174.2701	0.25644	149.3696
0.93492	189.9738	0.567177	171.6537	0.216783	144.7121
0.888576	187.979	0.521656	169.2313	0.176984	140.0031
0.842211	185.8907	0.477119	166.5707	0.139234	134.9253
0.795606	183.6965	0.432521	164.0158	0.101865	129.8898
0.750065	181.6302	0.388988	161.1396	0.087629	126.0205
0.704222	179.1816	0.342724	157.9746		
0.658179	176.7446	0.298126	153.884		

Table S2. Tabular data for the Ar adsorption isotherm of **ActB**(B_9-250˚C)

**Adsorption**					
P/P_0	V_{ads} (cm^3/g STP)	P/P_0	V_{ads} (cm^3/g STP)	P/P_0	V_{ads} (cm^3/g STP)
0.000162	9.30725	0.059698	94.79998	0.501014	130.0238
8.27E-05	18.82797	0.078733	98.52106	0.5478	132.2883
0.000262	28.15033	0.098211	101.4266	0.586534	134.1149
0.000586	37.31392	0.120078	103.7575	0.624244	135.6958
0.001249	46.23357	0.162306	107.8569	0.661974	137.365
0.002522	54.65774	0.206562	111.1909	0.698881	138.9758
0.004589	62.48288	0.249433	115.2433	0.734563	140.6149
0.007841	69.37459	0.292082	118.3167	0.782976	142.4621
0.013132	74.74196	0.334531	121.1119	0.832272	144.4064
0.025823	83.73038	0.37688	123.606	0.87968	146.3557
0.041565	90.29804	0.419449	125.8871	0.928535	148.2951
**Desorption**					
P/P_0	V_{ads} (cm^3/g STP)	P/P_0	V_{ads} (cm^3/g STP)	P/P_0	V_{ads} (cm^3/g STP)
0.976927	150.4586	0.614084	135.2699	0.265175	116.3777
0.911688	147.6263	0.555751	132.6633	0.209092	111.4301
0.850966	145.175	0.496777	129.8213	0.154113	107.0616
0.79149	142.7979	0.438264	126.8957	0.094456	100.8664
0.732194	140.5061	0.380755	123.8137		
0.673018	137.847	0.321961	120.2842		

Table S3. Tabular data for the Ar adsorption isotherm of **ActB**(B_{11}-300˚C)

**Adsorption**					
P/P_0	V_{ads} (cm^3/g STP)	P/P_0	V_{ads} (cm^3/g STP)	P/P_0	V_{ads} (cm^3/g STP)
9.45E-06	9.20695	0.080219	130.0683	0.535551	170.0176
4.08E-05	18.43227	0.092107	132.7569	0.563101	171.6494
0.000125	27.60488	0.102749	134.444	0.589727	173.2519
0.000297	36.65485	0.134857	139.1983	0.617437	174.6528

0.000599	45.57632	0.168651	143.0017	0.643661	176.1018
0.001116	54.32384	0.201743	146.2983	0.66854	177.4897
0.001908	62.68112	0.235598	149.2146	0.695146	178.8869
0.003039	70.56432	0.269533	152.4314	0.718479	180.238
0.004589	77.96155	0.303829	154.9077	0.744503	181.5929
0.006587	84.76255	0.333306	156.9973	0.782434	183.5887
0.009048	90.94768	0.362904	159.2317	0.80667	184.9681
0.013233	95.04674	0.394871	161.4183	0.848014	186.9944
0.024357	106.9831	0.425011	163.6218	0.887231	189.078
0.037891	115.5548	0.452782	165.354	0.928957	191.3559
0.051324	121.4429	0.483122	166.9315	0.968936	193.7599
0.065942	126.2025	0.507499	168.7091		

Desorption

P/P_0	V_{ads} (cm^3/g STP)	P/P_0	V_{ads} (cm^3/g STP)	P/P_0	V_{ads} (cm^3/g STP)
0.968936	193.7599	0.61828	178.522	0.300215	158.8239
0.92486	192.3317	0.578201	176.5982	0.256541	154.9112
0.876387	190.437	0.540912	174.3597	0.216823	151.2539
0.82669	188.3356	0.503504	172.1492	0.18086	146.8754
0.782875	186.4595	0.465352	169.8842	0.146945	142.3255
0.739543	184.419	0.428043	167.5391	0.114054	137.1633
0.697977	182.448	0.391498	165.1365	0.08289	130.6723
0.657818	180.5031	0.355334	162.5531		

Table S4: Tabular data for the Ar adsorption isotherm of **ActB**(B_{11}-250˚C) after treatment with 0.5 M H_2SO_4.

Adsorption

P/P_0	V_{ads} (cm^3/g STP)	P/P_0	V_{ads} (cm^3/g STP)	P/P_0	V_{ads} (cm^3/g STP)
0.003994	13.13698	0.323568	40.83969	0.68868	64.19495
0.020642	15.25024	0.365655	43.10454	0.733459	66.78116
0.04518	19.85892	0.402301	45.26815	0.763317	69.05997
0.07024	21.53297	0.435152	47.80575	0.791791	70.24725
0.094657	22.66535	0.470814	49.68252	0.841448	72.18796
0.122407	24.74387	0.504327	52.28989	0.88952	74.25895
0.163069	28.84224	0.540812	53.78934	0.940021	76.7539
0.203952	32.47232	0.575209	55.9997	0.98516	79.75778
0.24385	35.13028	0.609144	59.26992		
0.283307	38.99102	0.643762	60.83127		

Desorption

P/P_0	V_{ads} (cm^3/g STP)	P/P_0	V_{ads} (cm^3/g STP)	P/P_0	V_{ads} (cm^3/g STP)
0.98516	79.75778	0.633461	60.36667	0.304592	39.96837
0.93741	76.64648	0.58555	56.99624	0.252766	36.00263
0.883737	74.00981	0.536997	53.63255	0.206823	32.66361

0.833557	71.87955	0.491034	51.25569	0.163169	28.85115
0.785205	69.97262	0.445111	48.32989	0.119957	24.78777
0.734262	66.84246	0.39959	45.1081	0.077047	21.84866
0.684363	63.87166	0.354872	42.52428		

Table S5: Tabular data for the Ar adsorption isotherm of **ActB**(B_9-250˚C) after treatment with 0.5 M H_2SO_4.

Adsorption					
P/P_0	V_{ads} (cm^3/g STP)	P/P_0	V_{ads} (cm^3/g STP)	P/P_0	V_{ads} (cm^3/g STP)
0.017289	0.22442	0.356017	5.70495	0.71366	12.72236
0.048071	0.81564	0.405313	6.47213	0.752173	13.79662
0.075681	1.23556	0.448123	7.36872	0.787855	14.73101
0.109516	1.71771	0.496154	8.14703	0.842754	16.41085
0.159856	2.40465	0.541776	9.0512	0.898736	18.59572
0.209233	3.20662	0.587217	9.97962	0.956024	21.86626
0.257685	4.07502	0.63071	10.8359		
0.307765	4.81905	0.671492	11.74507		
**Desorption**					
P/P_0	V_{ads} (cm^3/g STP)	P/P_0	V_{ads} (cm^3/g STP)	P/P_0	V_{ads} (cm^3/g STP)
0.956024	21.86626	0.620569	12.19321	0.283548	6.51181
0.897431	19.24752	0.567458	11.08202	0.228851	5.81083
0.840063	17.32658	0.514588	10.09542	0.178209	4.98558
0.783518	15.82168	0.462621	9.23598	0.12821	4.48451
0.729503	14.58746	0.411638	8.50076	0.074978	1.22487
0.67346	13.28372	0.341298	7.41224		

Table S6: Tabular data for the Ar adsorption isotherm of **ActB**(B_9-300˚C) after treatment with 0.5 M H_2SO_4.

Adsorption					
P/P_0	V_{ads} (cm^3/g STP)	P/P_0	V_{ads} (cm^3/g STP)	P/P_0	V_{ads} (cm^3/g STP)
0.010896	0.95035	0.337403	8.61744	0.687295	19.04023
0.036023	1.23513	0.375093	9.63031	0.717154	20.12554
0.059999	1.68702	0.412381	10.60492	0.752294	21.30217
0.083251	2.02444	0.449509	11.86632	0.781831	22.42795
0.104074	2.58047	0.485171	12.77766	0.807413	23.48545
0.143511	3.18423	0.52027	13.80848	0.856589	25.35783
0.182326	3.86619	0.554808	14.79684	0.903415	27.36835
0.221481	4.73873	0.587839	15.92576	0.949538	29.69312
0.259734	6.17052	0.623581	16.95442	0.99504	34.02497
0.298588	7.28541	0.652657	17.92735		

Desorption					
P/P_0	V_{ads} (cm^3/g STP)	P/P_0	V_{ads} (cm^3/g STP)	P/P_0	V_{ads} (cm^3/g STP)
0.99504	34.02497	0.64577	18.71332	0.309471	8.75044
0.953153	30.4704	0.604606	17.23315	0.263569	7.49049
0.90725	28.1634	0.562699	16.05918	0.220277	6.2673
0.861649	26.30665	0.521816	14.62114	0.179615	4.999
0.818276	24.62297	0.481014	13.44981	0.139897	3.92662
0.777574	23.30345	0.440714	12.40492	0.101183	2.99749
0.733981	21.72713	0.401176	11.35211	0.061605	1.71033
0.689022	20.14335	0.360735	10.1454		

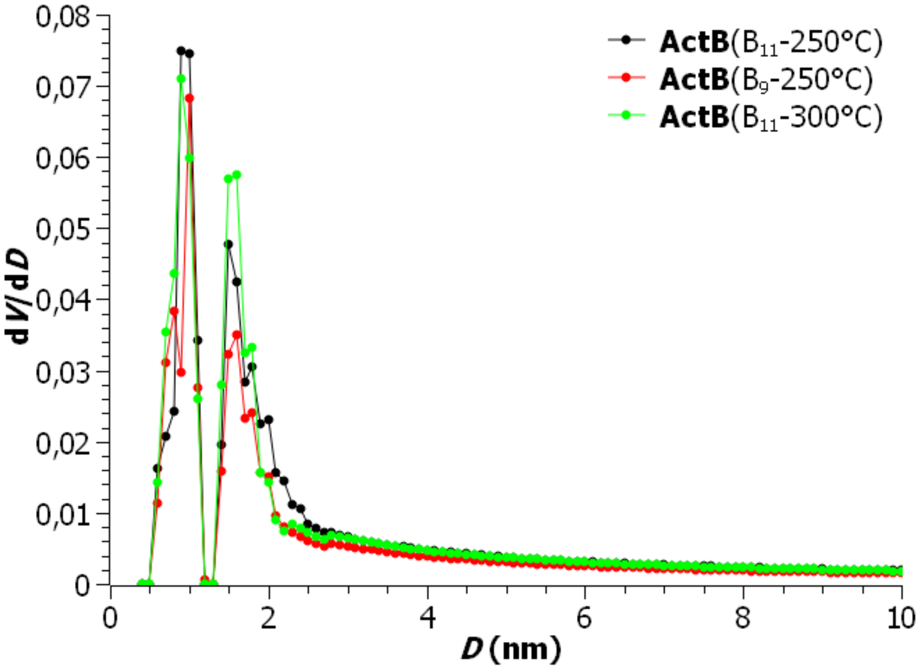

Figure S1. Pore size distribution for parent **ActB**s.

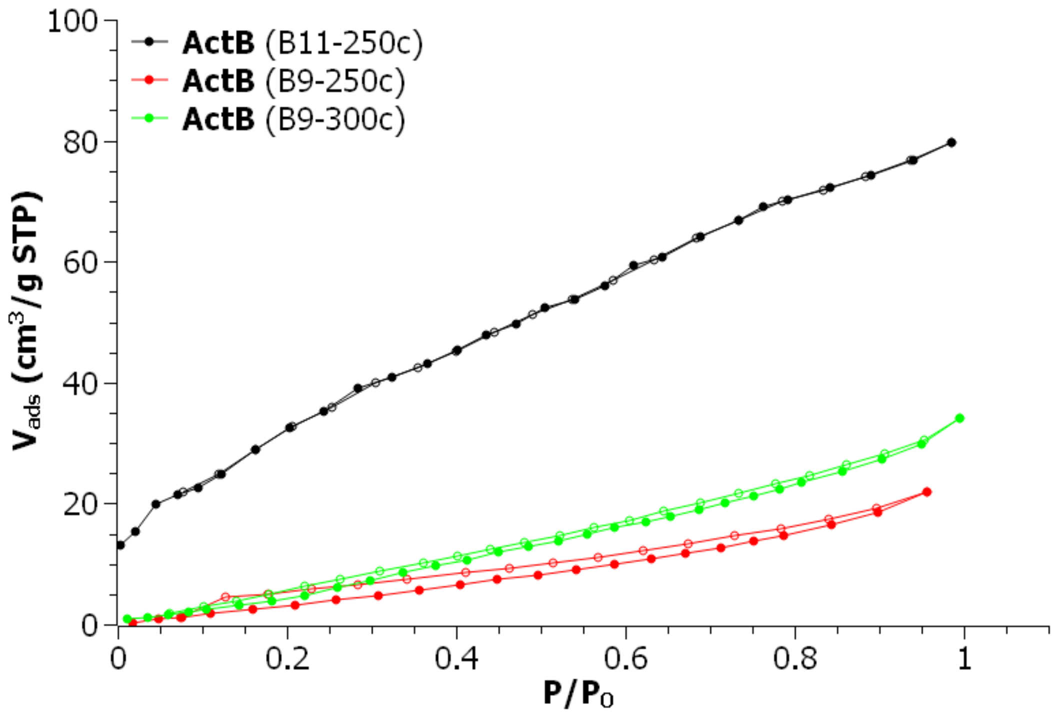

Figure S2. Adsorption isotherms of Ar (87 K) after treatment with 0.5 M H_2SO_4.

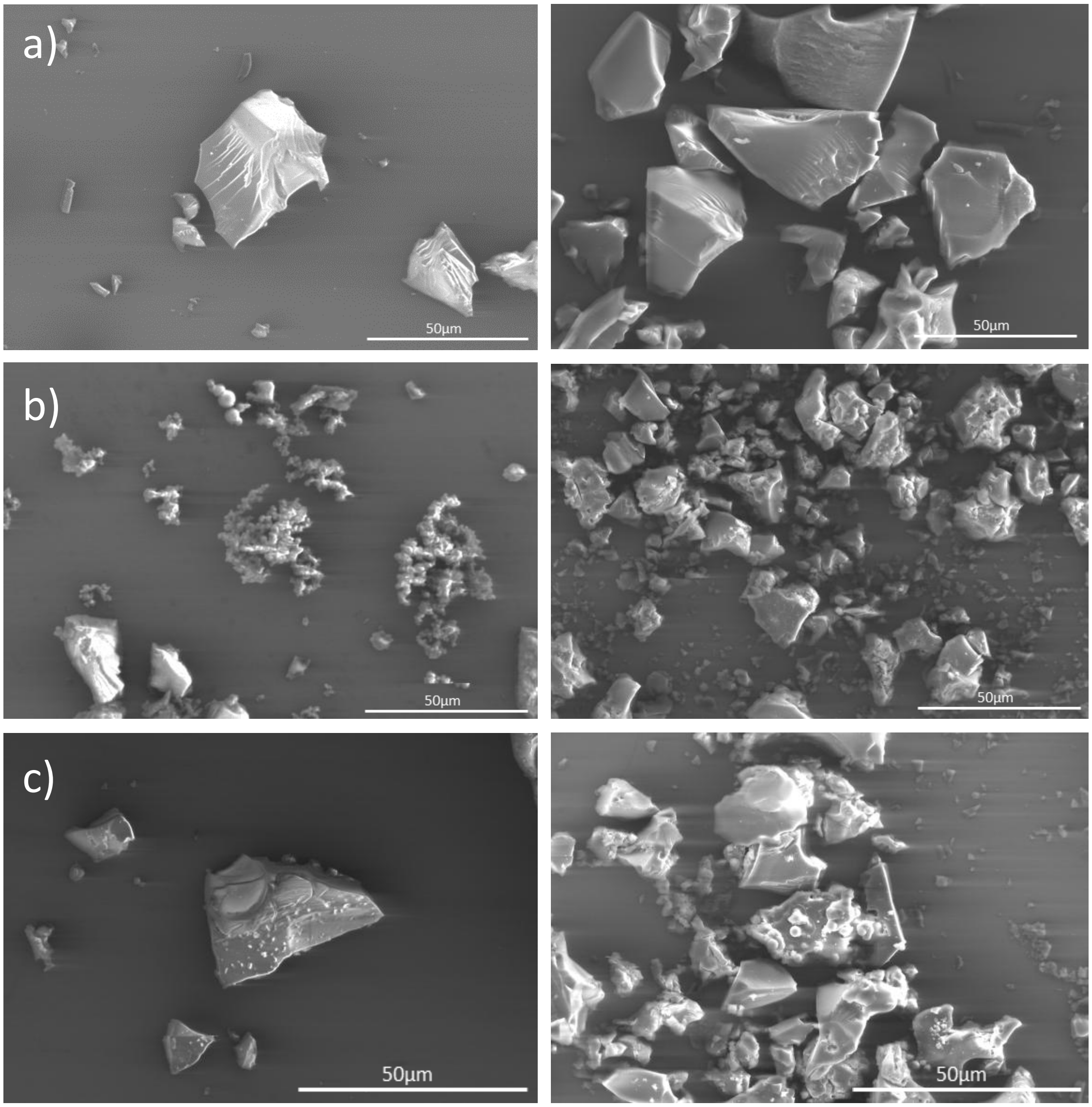

Figure S3 SEM images of **ActB**(B_{11}-250°C) (top), **ActB**(B_9-250°C) (middle), and **ActB**(B_9-300°C) (bottom). In all cases in the left column is parent **ActB** and in the right column after 0.5 M H_2SO_4 treatment.

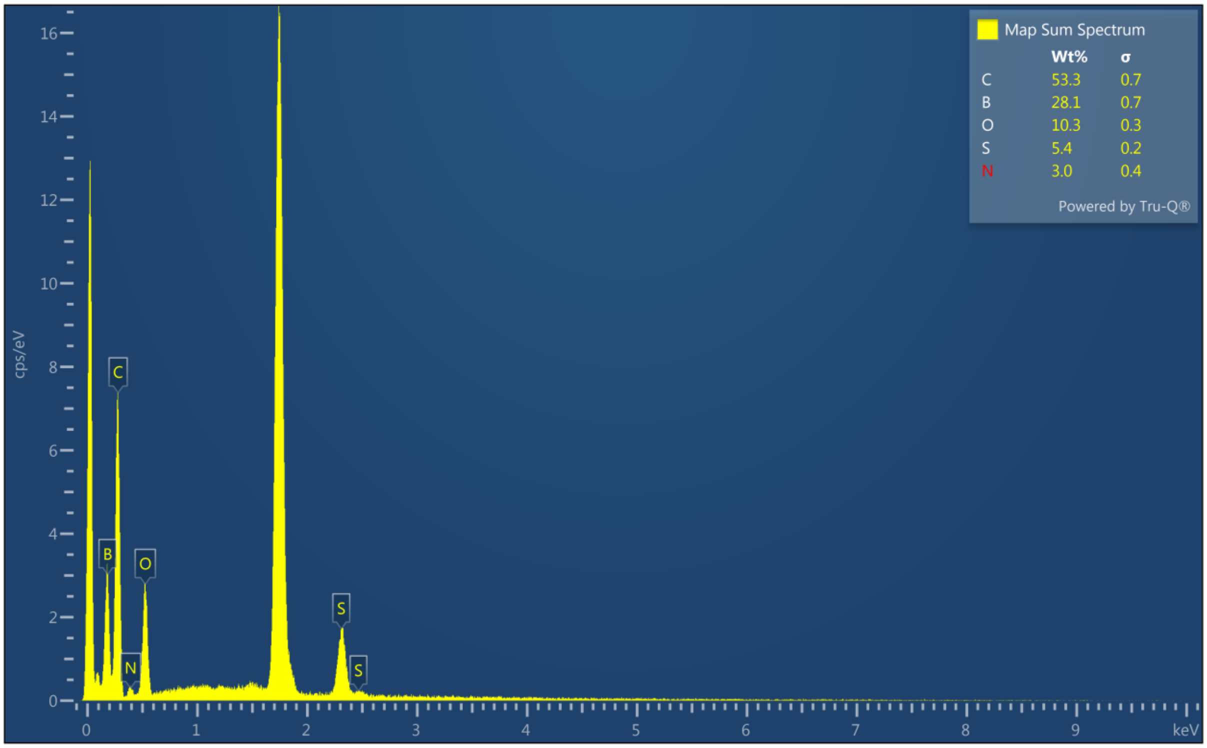

Figure S4. EDX of **ActB**(B_{11}-250°C) after treatment with 0.5 M H_2SO_4.

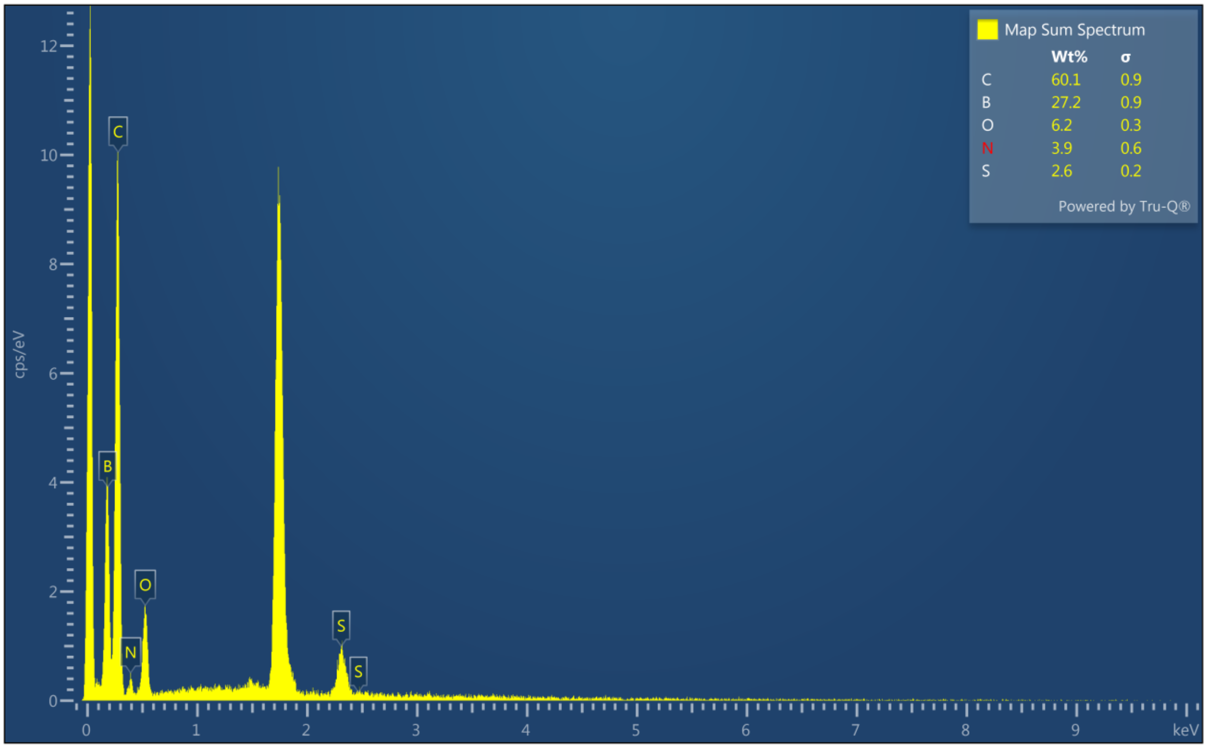

Figure S5. EDX of **ActB**(B_9-250°C) after treatment with 0.5 M H_2SO_4.

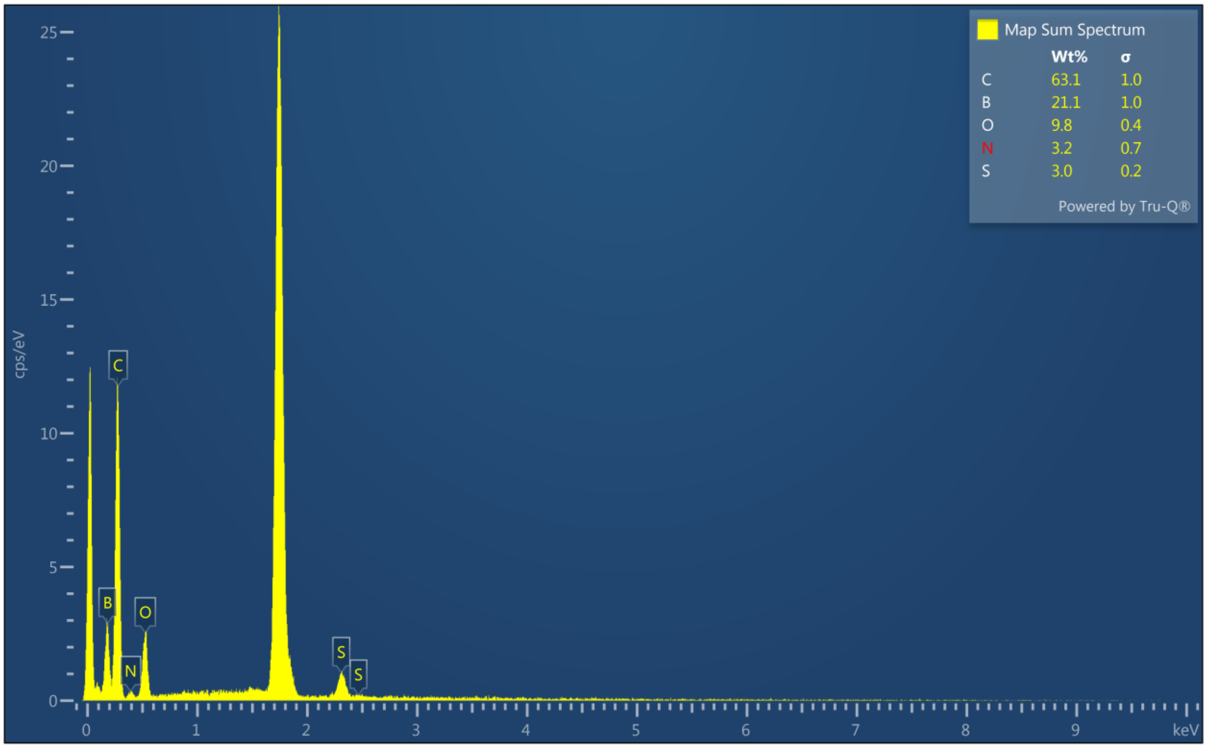

Figure S6. EDX of **ActB**(B_9-300°C) after treatment with 0.5 M H_2SO_4.

Table S7. Specific surface areas calculated from Ar adsorption at 87 K and nitrogen and sulphur content determined by EDX for **ActB**(B_{11}-250°C), **ActB**(B_9-250°C), and **ActB**(B_9-300°C) all after treatment in 0.5 M H_2SO_4 for 1 h at RT.

Sample	S_{BET} (m^2g^{-1})[a]	N (%)	S (%)
**ActB**(B_{11}-250°C)	86	3.0	5.4
**ActB**(B_9-250°C)	8	3.9	2.6
**ActB**(B_9-300°C)	18	3.2	3.0

[a] BET specific surface area.

Electrochemical SC measurements

The electrochemical performance was studied using the CHI electrochemical workstation 6273b (CHI-6273b) at room temperature in a typical three-electrode cell setup. The working electrode slurry was prepared via the dispersion of **ActB**s, carbon black, and polyvinylidene fluoride (PVDF) in a mass ratio of 80:10:10 in *N*-methyl-2-pyrrolidone as a solvent. Then, a 1*1 cm carbon cloth coated with the slurry was dried at 70 °C and used as a working electrode, and platinum wire and Ag/AgCl were used as counter and reference electrodes, respectively. Systems with H_2SO_4 and Na_2SO_4 electrolyte were fabricated by an analogous procedure. The Cyclic voltammetry (CV) and galvanostatic charge/discharge (GCD) experiments were measured at a range of -0.1 V to 0.5 V. Electrochemical impedance spectroscopy (EIS) was studied at a frequency range from 0.01 Hz to 100,000 Hz with an amplitude of 5 mV. The specific capacitance of the catalysts was calculated using the following equation.

$$C_p = \frac{I\Delta t}{m\Delta V} \qquad (1)$$

Where C_p (F g^{-1}) is the specific capacitance, I(A) is the applied current, Δt (s) is the discharge time, ΔV (V) is the potential window, and m (g) is the mass of active materials.

Fabrication of solid-state supercapacitor (SSC):

Asymmetric two-electrode supercapacitors have been established utilizing synthesized electrode materials to demonstrate their effectiveness. **ActB**(B_9-250°C) was utilized as a capacitance anode and a battery-type cathode/anode. The process described for the fabrication of the positive electrode also applies to the negative electrode (anode). The H_2SO_4/PVA gel electrolyte was prepared as follows: PVA and H_2SO_4 (1:1) were added to 27.0 g deionized water, followed by heating at 90 °C under magnetic stirring until the solution became clear. To fabricate the SCs,

asymmetric two-electrode supercapacitors through **ActB**(B_9-250°C) by way of the anode and cathode electrodes were fabricated, respectively. The fabricated electrodes were immersed in the H_2SO_4/PVA gel solution for 10 h, dried at room temperature until the gel electrolyte solidified, and then placed on Scotch tape in parallel. Finally, another piece of tape was pasted on them to form solid-state SCs. The electrochemical performances of the solid-state asymmetric two-electrode supercapacitors were evaluated using a two-electrode configuration on an electrochemical workstation (CHI 6273B). Each analysis was repeated at least three times to calculate the relative deviation and ensure that the data were comparable.

The mass proportion of the anode and cathode was calculated by equation 2,

$$\frac{m_+}{m_-} = \frac{C_- \times \Delta V_-}{C_+ \times \Delta V_+} \ldots\ldots\ldots\ldots\ldots(2)$$

where $m_\pm$, $C_{\pm,}$ and $\triangle V_\pm$ are the mass, potential window, and specific capacitance of the **ActB**(B_9-250°C) Asymmetric electrodes, respectively.

The energy density (E) (Wh/Kg) and power density (P) (W/Kg) were calculated in an assembled supercapacitor using the following equations,

$$E = C_s V^2/2 \qquad (3)$$

$$P = E/t \qquad (4)$$

Where C (C g^{-1}), ΔV (V), and Δt (s) are the specific capacity, discharge voltage, and discharge time of the hybrid capacitor device, respectively.

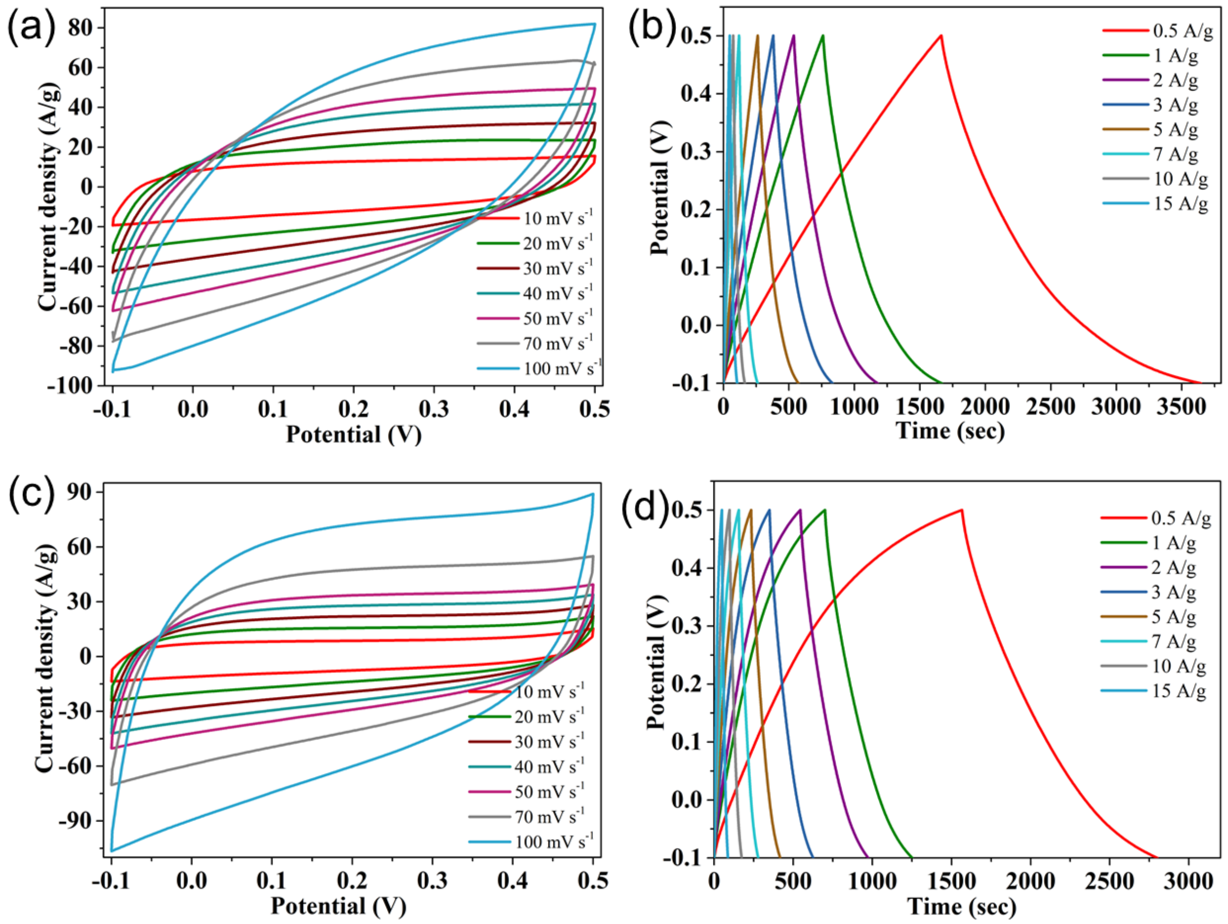

Figure S7. CV and GCD curves: (a-b) 1.0 M Na_2SO_4, and (c-d) 1.0 M KOH using **ActB**(B_9-250°C).

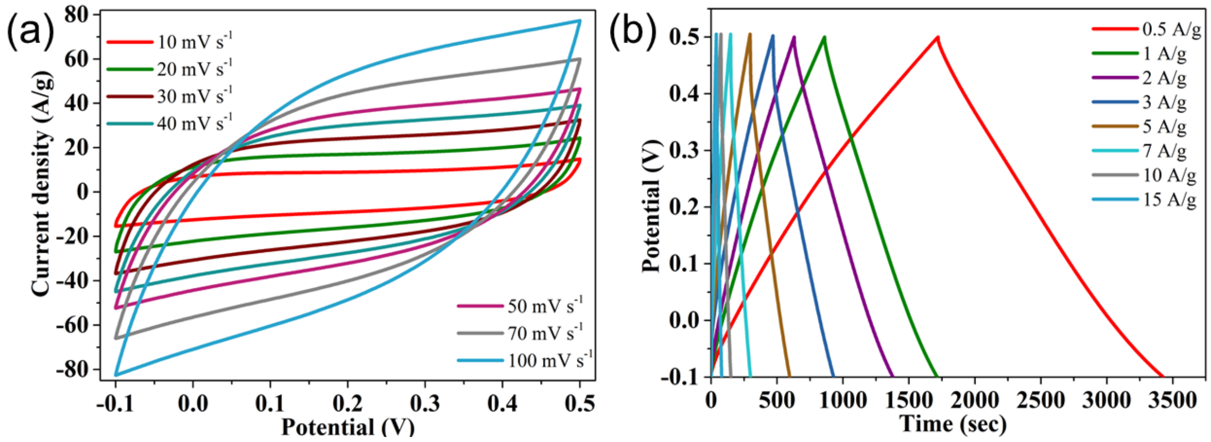

Figure S8. (a) CV curves and (b) GCD curves for **ActB**(B_{11}-250°C).

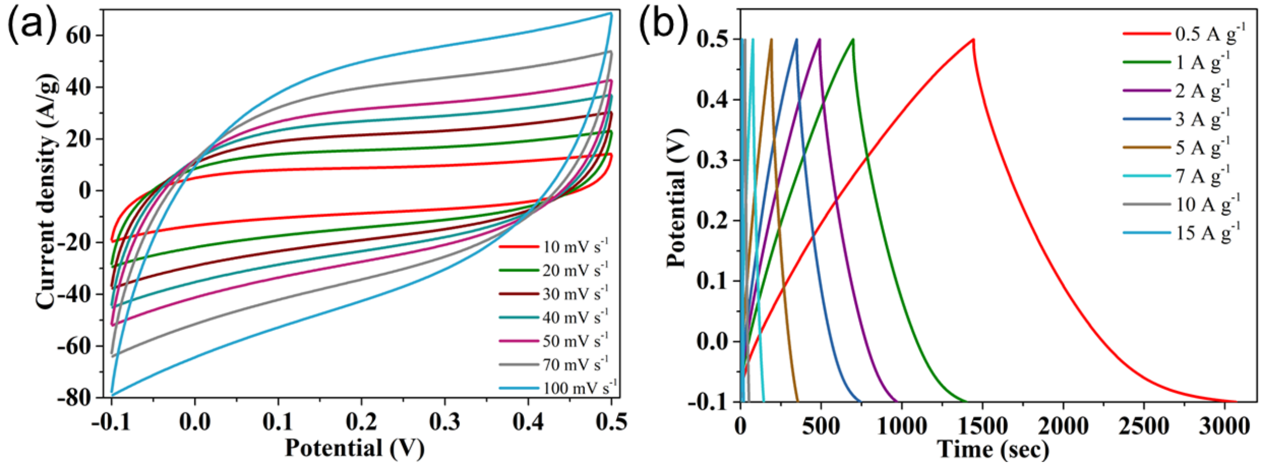

Figure S9. (a) CV curves and (b) GCD curves for **ActB**(B_9-300°C).

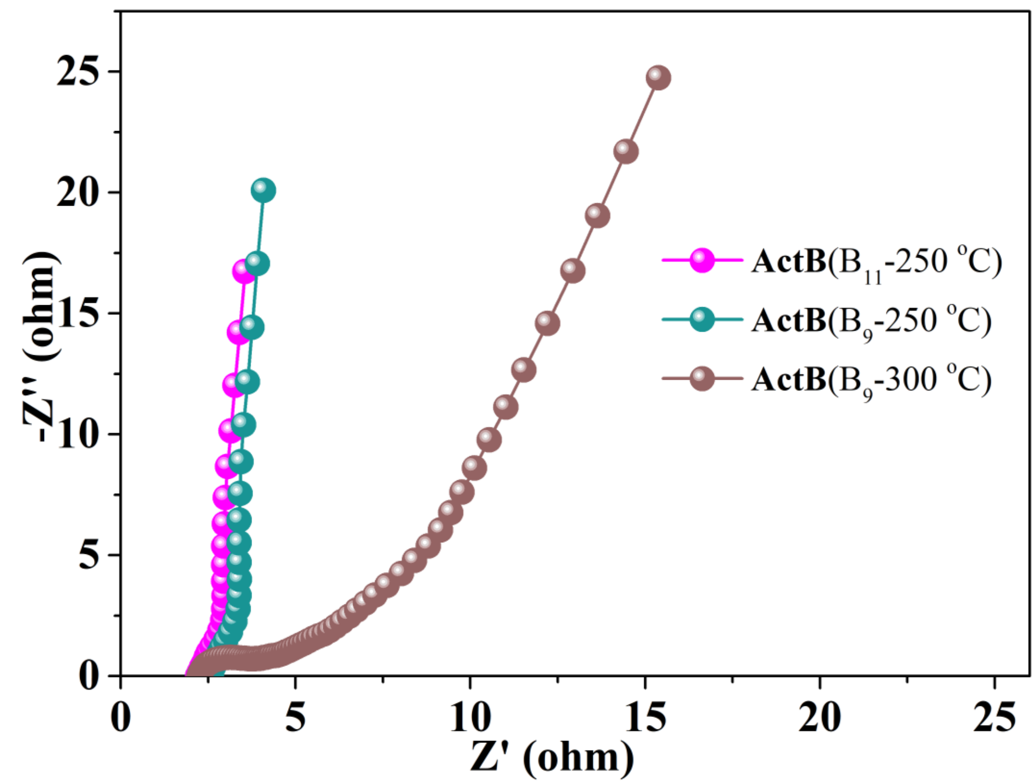

Figure S10. EIS Nyquist plots for **ActB**(B_9-250°C), **ActB**(B_{11}-250°C), and **ActB**(B_9-300°C).

Table S8. Comparison of electrochemical SC performance with previously obtained results.

Catalysts	Capacitance (F/g)	Retention (%)	Energy density (Wh/kg)	Power density (W/kg)	Ref.
**ActB**(B_9-250°C)	**607**	**95**	**25**	**486**	**This Work**
N, B co-doped GO (NB-GO)	525	77	23	872	6
BNDC/CP	504	97	22	200	7
3D N, B co-doped Graphene	239	86	8.7	1650	8
B/N co-doped carbon	423	100	6.9	80	9
$Co(OH)_2$-BN Doped Graphene	1263	90	20	9331	10
WPU-GO-Fe-B	330	90	7.9	505	11
CoB–AC	194	80	19	200	12
B, N-doped (BNAC-850) ACs	176	96	9.7	2000	13
B/S-Co-doped Carbon	290	70	16	248	14
B and N-doped graphene QDs	283	80	6.2	108	15
BNPC-X	286	92	-	-	16
polyaniline/B-doped graphene	406	83	20	382	17
Ni, Co-MOF@CW	1239	92	-	-	18
ANI@CNF/PVA	502	68	11.5	414	19

References:

1. O. L. Tok, J. Holub, A. Růžička, Z. Růžičková, B. Štíbr, (2018). Direct synthesis of dicarbollides. New Journal of Chemistry, *42, 2018, 8524–8529*. https://doi.org/10.1039/C8NJ00819A
2. S. G. Shore, in Boron Hydride Chemistry, ed. E. L. Muetterties, Academic, New York, 1975, pp. 79–174, and references therein
3. J.-P. Amoureux, C. Fernandez, S. Steuernagel, ZFiltering in MQMAS NMR, J. Magn. Res., Series A 123 (1996) 116–118. https://doi.org/10.1006/jmra.1996.0221.
4. A. Equbal, M. Bjerring, P.K. Madhu, N.Chr. Nielsen, Improving spectral resolution in biological solid-state NMR using phase-alternated rCW heteronuclear decoupling, Chem. Phys. Lett. 635 (2015) 339–344. https://doi.org/10.1016/j.cplett.2015.07.008.
5. J. Brus, Heating of samples induced by fast magic-angle spinning, Solid State Nucl. Magn. Reson. 16 (2000) 151–160. https://doi.org/10.1016/s0926-2040(00)00061-8.
6. D. Prakash, S. Manivannan, N, B co-doped and Crumpled Graphene Oxide Pseudocapacitive Electrode for High Energy Supercapacitor, Surfaces and Interfaces 23 (2021) 101025. https://doi.org/10.1016/j.surfin.2021.101025.
7. Z. Zhao, Y. Xie, Electrochemical supercapacitor performance of boron and nitrogen co-doped porous carbon nanowires, J. Power Sourc. 400 (2018) 264–276. https://doi.org/10.1016/j.jpowsour.2018.08.032.
8. Z. Wu, A. Winter, L. Chen, Y. Sun, A. Turchanin, X. Feng, K. Müllen, Three-Dimensional Nitrogen and Boron Co-doped Graphene for High-Performance All-Solid-State Supercapacitors, Adv. Mater. 24 (2012) 5130–5135. https://doi.org/10.1002/adma.201201948.

9. J. Hao, J. Wang, S. Qin, D. Liu, Y. Li, W. Lei, B/N co-doped carbon nanosphere frameworks as high-performance electrodes for supercapacitors, J. Mater. Chem. A 6 (2018) 8053–8058. https://doi.org/10.1039/c8ta00683k.
10. H. Tabassum, A. Mahmood, Q. Wang, W. Xia, Z. Liang, B. Qiu, R. zhao, R. Zou, Hierarchical Cobalt Hydroxide and B/N Co-Doped Graphene Nanohybrids Derived from Metal-Organic Frameworks for High Energy Density Asymmetric Supercapacitors, Sci. Rep. 7 (2017). https://doi.org/10.1038/srep43084.
11. R. Li, C. Qin, X. Zhang, Z. Lin, S. Lv, X. Jiang, Boron/nitrogen co-doped carbon synthesized from waterborne polyurethane and graphene oxide composite for supercapacitors, RSC Adv. 9 (2019) 1679–1689. https://doi.org/10.1039/c8ra09043b.
12. J.-F. Hou, J.-F. Gao, L.-B. Kong, Liquid phase reduction synthesis of a cobalt boride–activated carbon composite with improved specific capacitance and retention rate as a new positive electrode material for supercapacitors, New J. Chem. 43 (2019) 14475–14484. https://doi.org/10.1039/c9nj02830g.
13. Q. Lu, Y. Xu, S. Mu, W. Li, The effect of nitrogen and/or boron doping on the electrochemical performance of non-caking coal-derived activated carbons for use as supercapacitor electrodes, New Carbon Materials 32 (2017) 442–450. https://doi.org/10.1016/s1872-5805(17)60133-1.
14. Y. Wang, D. Wang, Z. Li, Q. Su, S. Wei, S. Pang, X. Zhao, L. Liang, L. Kang, S. Cao, Preparation of Boron/Sulfur-Codoped Porous Carbon Derived from Biological Wastes and Its Application in a Supercapacitor, Nanomaterials 12 (2022) 1182. https://doi.org/10.3390/nano12071182.

15. M.T. Dejpasand, S. Sharifi, E. Saievar-Iranizad, A. Yazdani, K. Rahimi, Boron- and nitrogen-doped graphene quantum dots with enhanced supercapacitance, J. Energy Storage 42 (2021) 103103. https://doi.org/10.1016/j.est.2021.103103.
16. L. Bai, Y. Ge, L. Bai, Boron and Nitrogen Co-Doped Porous Carbons Synthesized from Polybenzoxazines for High-Performance Supercapacitors, Coatings 9 (2019) 657. https://doi.org/10.3390/coatings9100657.
17. Q. Hao, X. Xia, W. Lei, W. Wang, J. Qiu, Facile synthesis of sandwich-like polyaniline/boron-doped graphene nano hybrid for supercapacitors, Carbon 81 (2015) 552–563. https://doi.org/10.1016/j.carbon.2014.09.090.
18. Y. Zhang, C. Xiong, Q. Xiong, Q. Xiong, M. Zhao, B. Wang, M. Shen, Q. Zhou, Y. Ni, Ni, Co bimetallic MOF of dual-controlled by micro-morphology and unit cell structure for biomass-based self-supporting energy storage device, Rare Met. 44 (2025) 8536–8547. https://doi.org/10.1007/s12598-025-03506-5.
19. C. Xiong, C. Zheng, Z. Zhang, Q. Xiong, Q. Zhou, D. Li, M. Shen and Y. Ni, Polyaniline@cellulose Nanofibers Multifunctional Composite Material for Supercapacitors, Electromagnetic Interference Shielding and Sensing. J. Materiomics, 2025, 11 (1), 100841. https://doi.org/10.1016/j.jmat.2024.01.015.
